# A novel LGALS1-depended and immune-associated fatty acid metabolism risk model in acute myeloid leukemia stem cells

**DOI:** 10.1038/s41419-024-06865-6

**Published:** 2024-07-05

**Authors:** Huanhuan Qin, Meixi Peng, Jingsong Cheng, Zhenyu Wang, Yinghui Cui, Yongxiu Huang, Yaoqi Gui, Yanni Sun, Wenqiong Xiang, Xiaomei Huang, Ting Huang, Li Wang, Jieping Chen, Yu Hou

**Affiliations:** 1 The First Clinical Institute, Zunyi Medical University, Zunyi, 563006 China; 2 Department of Radiological Medicine, School of Basic Medical Sciences, Chongqing Medical University, Chongqing, 400016 China; 3 The Second Clinical College, Chongqing Medical University, Chongqing, 400016 China; 4 Guizhou Provincial College-Based Key Lab for Tumor Prevention and Treatment with Distinctive Medicines, Zunyi Medical University, Zunyi, 563006 China; 5 Department of Hematology/Oncology, Children’s Hospital of Chongqing Medical University, Chongqing, 400014 China; 6 Department of Hematology, Southwest Hospital, Third Military Medical University (Army Medical University), Chongqing, 400038 China; 7 Medical School of Guizhou University, Guiyang, 550025 China; 8 Department of Hematology, The First Affiliated Hospital of Chongqing Medical University, Chongqing, 400016 China; 9 Obstetrics and Gynecology Department, The First Affiliated Hospital of Chongqing Medical University, Chongqing, 400016 China; 10 Department of Gynecology and Obstetrics, Chongqing Health Center for Women and Children, Women and Children’s Hospital of Chongqing Medical University, Chongqing, 400016 China; 11 Chongqing Key Laboratory of Hematology and Microenvironment, Chongqing Medical University, Chongqing, 400016 China

**Keywords:** Cancer stem cells, Cancer metabolism

## Abstract

Leukemia stem cells (LSCs) are recognized as the root cause of leukemia initiation, relapse, and drug resistance. Lipid species are highly abundant and essential component of human cells, which often changed in tumor microenvironment. LSCs remodel lipid metabolism to sustain the stemness. However, there is no useful lipid related biomarker has been approved for clinical practice in AML prediction and treatment. Here, we constructed and verified fatty acid metabolism-related risk score (LFMRS) model based on TCGA database *via* a series of bioinformatics analysis, univariate COX regression analysis, and multivariate COX regression analysis, and found that the LFMRS model could be an independent risk factor and predict the survival time of AML patients combined with age. Moreover, we revealed that Galectin-1 (LGALS1, the key gene of LFMRS) was highly expressed in LSCs and associated with poor prognosis of AML patients, and LGALS1 repression inhibited AML cell and LSC proliferation, enhanced cell apoptosis, and decreased lipid accumulation in vitro. LGALS1 repression curbed AML progression, lipid accumulation, and CD8^+^ T and NK cell counts in vivo. Our study sheds light on the roles of LFMRS (especially LGALS1) model in AML, and provides information that may help clinicians improve patient prognosis and develop personalized treatment regimens for AML.

## Introduction

Acute myeloid leukemia (AML) is the most common and lethal adult acute leukemia characterized by the impaired differentiation of myeloid progenitor cells and clonal expansion of immature myeloid cells [1]. It has been reported that the mortality rate caused by leukemia has reached 3–4%, which is one of the main causes of cancer death [2]. Although many significant breakthroughs have been achieved in AML chemotherapy, targeted therapy, and immunotherapy, such as cytarabine + anthracycline (7 + 3) intensive chemotherapy, IDH1/2 inhibitors, and PD-1 blockade, postremission relapses occur frequently [3–5]. Growing evidences indicate that AML as well as other malignancies are maintained with a minor subpopulation referred to as leukemia stem cells (LSCs), which are recognized as the root cause of leukemia initiation, relapse, and drug resistance [6, 7]. Thus, exploring the AML features, especially the LSC features, is urgently needed, so as to exploit novel therapies.

The aberrant metabolisms in tumor microenvironment (TME) for tumor cells caused by nutrient deficiency and hypoxia facilitates tumor metastasis, proliferation, and survival [8]. Lipid species are highly abundant and essential component of human cells, which often changed in tumor disease [9]. Leukemia cells, especially LSCs, often show lipid metabolic abnormality, which has been the novel field and attracting extensive interests in the last several years [10]. Such as, m^6^A reader IGF2BP2-mediated lipid transporter MFSD2A has a critical role in LSCs function [11]. LSCs evade chemotherapy by absorbing fatty acid in microenvironment [12]. Unfortunately, owing to insufficient prospective and validated research works, no useful lipid related biomarker has been approved for clinical practice in AML treatment. Thus, there is an urgent need to identify efficient and complementary prognostic parameters and risk classification methods about lipid metabolism and LSCs.

AML has been considered an immunoresponsive malignancy [13]. Recent years, increasing of immunotherapy for AML has appeared, such as antibody therapy [14] (targeting CD33, CD123, and several other antigens), redirected T cells [14, 15] (anti-CD19 CAR T cells, anti-CD33 CAR T cells, anti-CD123 CAR T cells, et al.), checkpoint inhibitors (targeting PD-1/PD-L1) [16]. While impressive progress has been made in the clinical application of immunomodulatory agents, there are still some problems, such as CD33 and CD123 also expressed in normal hematopoietic cells, some AML patients not sensitive to anti-PD-1/PD-L1 [17]. It is reported that lipids threaten an anti-tumor environment whereby metabolic adaptation to lipid metabolism is linked to immune dysfunction [18]. Thus, we also combined lipid metabolism, immune status, and LSC stemness in our study.

Above all, we aimed to shed more light on the possible significance of fatty acid metabolism-related risk score (LFMRS) model in stratifying AML patient prognosis and its feasibility to guide therapeutic selection. Additionally, we also analyzed the correlation between LFMRS model and immune statuses. The prognostic model was constructed based on the Cancer Genome Atlas (TCGA) database, followed by further validation using BeatAML database and other GEO databases. Based on this, we further certificated Galectin-1 (LGALS1, the key gene in LFMRS model) plays a key role in lipid metabolism reprogramming, immunosuppressive effect, AML progress in vitro and in vivo. Our study sheds light on the roles of LFMRS model, especially LGALS1, in AML and provides information that may help clinicians improve patient prognosis and develop personalized treatment regimens for AML.

## Materials and methods

### Data acquisition and identification of the fatty acid metabolism-related genes

We collected acute myeloid leukemia (AML) samples and corresponding clinical data from different databases. Specifically, we obtained RNA-seq data and matched clinical information of 151 AML samples from The Cancer Genome Atlas (TCGA) through UCSC Xena (https://xenabrowser.net/). Additionally, we utilized AML samples from the Gene Expression Omnibus (GEO) database (GSE12417, GSE71014, and GSE37642), Therapeutically Applicable Research to Generate Effective Treatments (TARGET) database, and Beat AML database. The data forms of TCGA, TARGET, and BeatAML were transformed from fragments per kilobase of transcript per million fragments mapped (FPKM) to transcripts per kilobase million (TPM), then which were transformed using log2(TPM + 1). Fatty metabolism-related gene sets of Hallmark, KEGG, Reactom and Wikipathway were collected from Molecular Signatures (MsigDB) database (https://www.gsea-msigdb.org/). The single cell RNA-seq data was obtained from the GEO database using accession number GSE116256.

### Collection and enrichment Analysis of differentially expressed genes between HSCs and LSCs

The differentially expressed genes (DEGs) of HSCs and LSCs were analyzed by “limma” R package in GSE68172, GSE17054, and GSE24395 with a *p* value < 0.05 and |logFC | > 1 were used to identify the DEGs. Moreover, Robust Rank Aggregation (RRA) was used to further identify robust DEGs. The Single Sample GSEA (ssGSEA) analysis was completed by “GSVA” R package. A total of 211 robust differentially expressed genes were enriched, which including 109 up-regulated genes and 102 downregulated genes, and pathway enrichment for differentially expressed genes was performed by Metascape.

### Clinical samples

The bone marrow samples of 18 newly diagnosed AML patients and 13 healthy donors were obtained from the First Affiliated Hospital of Chongqing Medical University. Importantly, healthy samples were acquired from age-matched donors according to AML patients. All patients were informed and consented to participate in the study. The patients’ clinical characteristics are presented in detail in Table 1 and Table 2.

**Table 1:** Clinical characteristics of newly diagnosed patients.

Characteristics	Median(range)	All cases
Sex		
Female		17
Male		14
Total		31
Median age, y	54(31–80)	
Younger than 40 y		7
40–60 y		12
Older than 60 y		12
Median WBC, 10^9^/L	4.16 (0.9–186.53)	
Median platelets, 10^9^/L	38 (5–376)	
Iron deficiency anemia		13
AML		18
AML FAB subtype		
AML without maturation:M1		1
AML with maturation:M2		7
Acute promyelocytic leukemia:M3		2
Acute myelomonocytic leukemia:M4		4
Acute monoblastic or monocytic leukemia:M5		3
unclassified		1
Gene mutations		
NPM1		2
FLT3/ITD		4
WT1		7
DNMT3A		2

*AML* acute myeloid leukemia, *y* year old, *WBC* white blood cell, *FAB classification* French-American-British classification, a classification of acute leukemia produced by three-nation joint collaboration.

**Table 2:** The detailed gene mutations and FAB subtype in patients with AML.

Case NO.	Gene mutations	FAB subtype	Age
AML#1	KRAS/NRAS/FLT3-TKD/FLT3/GATA2/CEBPA/PML-RARA	M3	35
AML#2	MLL-AF9/WT1/EP300/TTN	M4	65
AML#3	PML-RARA(V)	M3	31
AML#4	FLT3-ITD/PTPN11/KMT2A/GATA2/CEBPA	M2	43
AML#5	/	M5	56
AML#6	TP53/NF1/FBXW7	M4	49
AML#7	WT1	M2	47
AML#8	NORMAL	/	51
AML#9	WT1/ZRSR2/NRAS/TET2/ETV6/SH2B3/KMT2A	M4	69
AML#10	IDH2/KRAS/DNMT3A/MPL/WT1	M5	74
AML#11	TP53/BCOR/NOTCH2/CYP2C19	M2	53
AML#12	BCOR/KRAS/RUNX1/TET2/ASXL1/BCORL1/SH2B3	M2	70
AML#13	WT1/ZRSR2/NPM1/TET2/GATA2	M1	38
AML#14	FLT3-ITD/DNMT3A/IDH1/WT1/	M5	78
AML#15	WT1/CEBPA/NPM1/IDH1/TET2/SH2B3	M4	32
AML#16	KIT/TET2	M2	71
AML#17	FLT3-ITD/PTPN11	M2	63
AML#18	TET2/ASXL1/BCORL1	M2	77

To obtain mononuclear cells from with AML patients and healthy control, we isolated mononuclear cells from bone marrow aspirates *via* density gradient centrifugation with using Ficoll mononuclear cell separation solution (#P8900, Solarbio, China). Finally, total mRNA and proteins were isolated for the analysis of LGALS1 expression. To obtain HSCs from mononuclear cells of healthy control, we isolated Lin^-^CD34^+^CD38^-^ cells [19] via flow cytometric analysis using anti-CD34-APC (1:100, BioLegend, America), anti-CD38-PE (1:100, BioLegend, America) after depleting human-lineage-positive cells using EasySep^TM^ Human Progenitor Cell Enrichment Kit II (#17936, STEMCELL^TM^ TECHNOLOGIES, Canada) [20]. To obtain LSCs from mononuclear cells of AML patients, we isolated CD34^+^CD38^-^ cells [21] via FCM using anti-CD34-APC (1:100, BioLegend, America), anti-CD38-PE (1:100, BioLegend, America).

### Cell culture

Human acute myeloid leukemia cell lines K562, HEL, KG-1, THP1, KG-1a, HL60, NB4, MV4-11, and U937 were obtained from the American Type Culture Collection (ATCC, America). OCI-AML3 was purchased from Deutsche Sammlung von Mikroorganismen und Zellkulturen GmbH (DSMZ, Germany). All cell lines were tested free of mycoplasma contamination and authenticated by the short tandem repeat (STR)-based method. The myeloid leukemia cell lines (K562, HEL, KG-1, THP1, KG-1a, HL60, NB4, MV4-11, and U937) were cultured in RPMI-1640 medium (#8123172, Gibco, America) supplemented with 10% of fetal bovine serum (FBS; #C04001-050×10, VivaCell, Israel) and 1% of penicillin/streptomycin solution (#15140-122, Gibco, America). OCI-AML3 was maintained in alpha-MEM (#32571101, Thermo Fisher Scientific, Germany) supplemented with 10% FBS and 1% of penicillin/streptomycin solution. LSCs were maintained in StemSpan^TM^ SFRM II (#09720, STEMCELL Technologies, Canada) plus 350 μm/L UM729 (#72332, STEMCELL Technologies), 1 m/L SR1 (#72354, STEMCELL Technologies). All cell lines were incubated in a humidity chamber (Thermo 371, Thermo Fisher Scientific) containing 5% CO_2_ at 37 °C.

### Reverse transcription PCR and quantitative real-time PCR (qRT-PCR)

Total RNA samples were obtained using TRIzol reagent (#AM91951A, TaKaRa, Japan) according to the manufacturer’s instructions [22]. cDNA was reverse transcribed with the use of the Prime-ScriptTM RT Reagent Kit (#AMG1420A, TaKaRa), and subjected to qRT-PCR analysis in the CFX Connect Real-Time PCR Detection System (#1855201, BIO-RAD, America). GAPDH was detected as an internal control for the indicated genes. The sequences of the primers used are shown in Table 3. All experiments were performed at least three times.

**Table 3:** The primer sequences used for qRT-PCR.

Gene	Forward (5ʹ-3ʹ)	Reverse (5ʹ-3ʹ)
LGALS1	TCGCCAGCAACCTGAATCTC	GCACGAAGCTCTTAGCGTCA
CD36	GGCTGTGACCGGAACTGTG	AGGTCTCCAACTGGCATTAGAA
PPAR-γ	TTTTCAAGGGTGCCAGTTTC	TTATTCATCAGGGAGGCCAG
ELOVL7	GCCTTCAGTGATCTTACATCGAG	AGGACATGAGGAGCCAATCTT
ALDH1A1	GCACGCCAGACTTACCTGTC	CCTCCTCAGTTGCAGGATTAAAG
ACOX2	GCACCCCGACATAGAGAGC	CTGCGGAGTGCAGTGTTCT
ACSM3	AGGAAGATGCTACGTCATGCC	ATCCCCAGTTTGAAGTCCTGT
GAPDH	GGAGCGAGATCCCTCCAAAAT	GGCTGTTGTCATACTTCTCATGG

### Western blotting

Western blotting was performed as previous described [23]. Briefly, total cellular proteins were acquired using RIPA lysis buffer (#P0013, Beyotime, China) and following with electrophoresed by 10% sodium dodecyl sulfate-polyacrylamide gel electrophoresis (SDS-PAGE), then incubated with primary antibodies against LGALS1 (1:1000, # A5590, Selleck, America), or GAPDH (1:1000, #ab8245, Abcam, Britain). GAPDH was detected as a loading control. The proteins were visualized with the enhanced chemiluminescence system (#01900MF, BIO-OI, China). All experiments were performed at least three times.

### Lentivirus production and transduction

To generate specific shRNA targeting LGALS1, we designed the primers for PCR of human LGALS1 coding sequences and the oligos for shRNAs. The shRNAs oligos were cloned into the vector pLKO.1-puro. The shRNA sequences were as follows: shNC, 5ʹ- CCGGTGCGCGATAGCGCTAATAATTTCAAGAGAATTATTAGCGCTATCGCGCTTTTTTG-3ʹ; shLGALS1#1, 5ʹ- CCGGGCTGCCAGATGGATACGAATTCTCGAGAATTCGTATCCATCTGGCAGCTTTTTG-3ʹ; shLGALS1#2, 5ʹ- CCGGCGCTAAGAGCTTCGTGCTGAACTCGAGTTCAGCACGAAGCTCTTAGCGTTTTTG-3ʹ; shLGALS1#3, 5ʹ- CCGGGTGTGTAACACCAAGGAAGATCTCGAGATCTTCCTTGGTGTTACACACTTTTTG-3ʹ.

For lentivirus transduction, the cells were infected with a lentivirus in the presence of 5 μg/mL polybrene (#C0351, Beyotime, China), and selected with 5 μg/mL puromycin (#ST551, Beyotime, China) for 7 days. The puromycin-resistant cells were collected for further analysis.

### Flow cytometry (FCM)

For Edu assay, Edu was added to HEL, THP1, or LSCs to a final concentration with 10 μM. After incubation for 2 h, the cells were washed twice with phosphate buffer saline (PBS), and stained according to the manufacturer’s instructions using Click-iT Plus EdU Alexa Fluor 647 Flow Cytometry Assay Kit (#C10634, Invitrogen, America). For Ki67 assay, HEL, THP1, or LSCs were stained with anti-ki67-PE antibody (#350503, Biolegend, America) for 30 min at 4 °C, before being stained with 4′,6-diamidino-2-phenylindole (DAPI) (10 μg/mL) overnight. For detecting apoptosis, HEL, THP1, or LSCs were stained with anti-Annexin V-APC (#640919, Biolegend, America) and DAPI in a binding buffer with Ca^2+^.

Flow cytometric analysis was done by gating on single cells and dead cells were excluded by DAPI staining. Data was analyzed using FlowJo Version 10 software (TreeStar, Ashland, OR, USA).

### Colony-forming assay

To determine cell proliferation ability, Colony-forming assay was performed. About 1 × 10^3^ AML cells were plated in a 12 well-plate containing Methylcellulose Complete Media (R&D Systems). After 7 days of incubation, colony numbers were scored. The colony-forming units were counted using an inverted microscope (#8THUNDER13, Leica, Germany).

### Oil Red O staining

The AML cells were counted and plated into a six-well plate (1 × 10^6^/ cell per well). 24 h later, Oil Red O (#C0157S, Beyotime, China) staining was performed as manufacturer’s instructions. Briefly, after washing with PBS, the cells were fixed with 4% paraformaldehyde for 10 min. Next, adding appropriate amount of dying washing solution to cover cells for 20 s, then adding appropriate amount of oil red O dying solution at room temperature for 30 min. After washing with dying washing solution and PBS, the cells were observed under a microscope (Ni-U, Nikon, Japan).

### Animal experiments

The female NOD-SCID IL2Rgnull (NSG) (6-8 weeks old) and C57BL/6 (6-8 weeks old) mice were purchased from Shanghai Southern Model Biotechnology Co., Ltd., and raised in the Experimental Animal Center of Chongqing Medical University. All mice were raised in accordance with the Association for Nursing Assessment and Accreditation of Experimental Animals and NIH standards. Importantly, mice were randomly assigned to one of groups. The experiment was approved by the Committee for the Management and use of Experimental Animals of Chongqing Medical University.

To verify the inhibitory effect of OTX008 on AML in mice, we constructed MLL-AF9 induced AML mouse model. The bone marrow cells of wild type mice were taken out after injection of 5-Fu, and then infected with MLL-AF9-GFP virus. Then the infected cells were transplanted into C57BL/6 J recipient mice through tail vein (irradiated in advance). The peripheral blood chimerism rate of mice was detected every week after transplantation. When the peripheral blood chimerism rate reached 50%-80%, the mice were killed and GFP^+^ leukemia cells from bone marrow were sorted for the second round of transplantation. After AML model mice were constructed, the peripheral blood chimerism rate of mice was detected every week after transplantation. When the proportion of GFP^+^ cells was about 10%, OTX008 (50 mg/kg,) was injected *via* intraperitoneal injection.

To verify the inhibitory effect of shLGALS1 on AML in mice, HEL-induced AML mouse model was constructed. HEL cells infected by LGALS1-shNC-GFP and LGALS1-sh#2-GFP virus were sorted by flow cytometry, and the same number of GFP^+^ cells (2 × 10^6^/mouse) were transplanted into NSG mice through tail vein. The proportion of GFP^+^ in peripheral blood of mice was detected by flow cytometry every week after transplantation.

The general appearance of the mice was observed every other day. The Kaplan-Meier survival analysis was used to evaluate the survival of the mice. Immature cells from BM were assessed by Wright’s staining. The infiltration of leukemic cells in liver and spleen was analyzed through hematoxylin and eosin (H&E) staining.

### Statistical analysis

R project 4.2.1, SPSS29.0 and GraphPadPrism8.0 statistical software were used to analyze the data and draw the graph. Unsupervised clustering of the patient samples at the different molecule levels were performed with the R package “ConsensusClusterPlus”. LASSO regression algorithm was used to screen genes related to fatty acid metabolism in differentially expressed genes in HSCs and LSCs by R package “glmnet”, and univariate COX regression analysis and multivariate COX regression analysis were used to determine independent prognostic factors. To better predict the prognosis, a nomogram analysis based on independent prognostic factors was established. The prognostic value was verified by Kaplan-Meier survival analysis using R package “survival”. Chi-square test was used to compare the clinical features of the two groups, and double-tailed t-test was used to analyze the quantitative difference between the two groups. ROC curve and survival curve were plotted by GraphPadPrism8.0 statistical software, and *p* < 0.05 was taken as statistical significance. For experiments, data from three independent experiments are shown as the mean ± standard deviation (SD). One-way analysis of variance was utilized to assess the differences between three or more groups. Unpaired Student’s t-test was used to compare the differences between the two groups. To compare survival differences, both the Kaplan-Meier estimator and log-rank test were utilized. Statistical analyses were performed using GraphPad Prism 8.0. (*p < 0.05, **p < 0.01, ***p < 0.001, ****p < 0.0001, and ns indicates no significant difference). The sample sizes were determined by referring recent papers. The in vitro sample size was completed according to enable statistical analyses.

## Result

### Identification of subtypes and construction of the LFMRS in AML

**Fig. 1:**
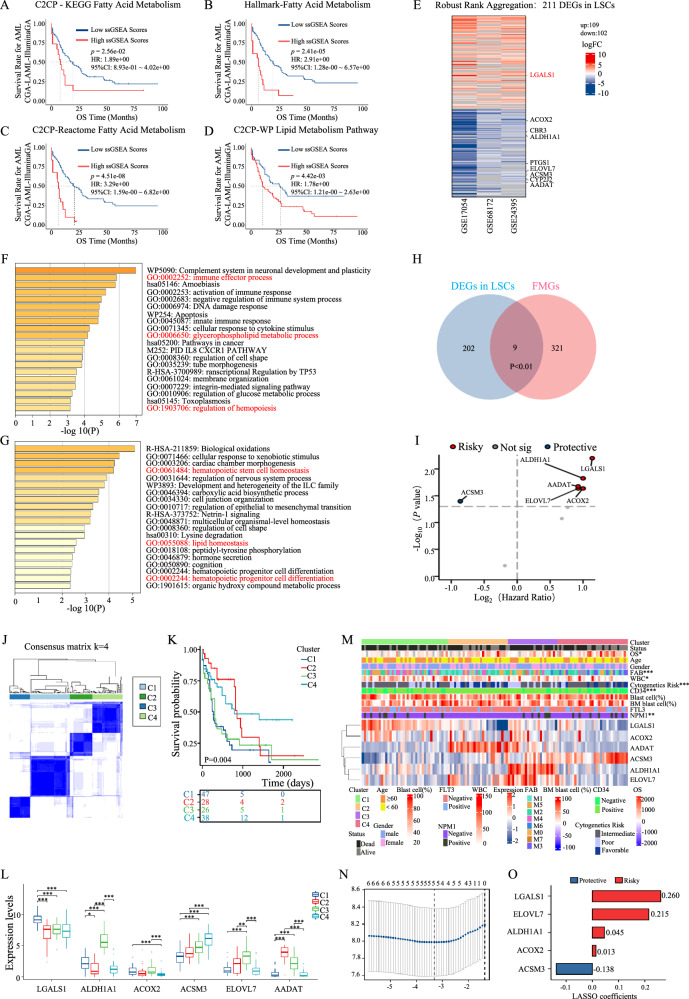
Identification of subtypes and construction of the LFMRs in AML. The survival analyses of ssGSEA score of fatty acid metabolism-related gene sets from KEGG (**A**), Hallmark (**B**), Reactom (**C**) and Wp (**D**) in the TCGA-LAML cohort. **E** The differential expression genes between LSCs and HSCs in the three databases (GSE17054, GSE68172, GSE24395) were shown in a heatmap. Red represents the significantly upregulated genes in LSCs compared with HSCs. Blue represents the significantly downregulated genes in LSCs compared with HSCs. Function and pathway enrichment analysis of the significantly upregulated genes (**F**) and downregulated genes (**G**) in LSCs versus HSCs by Metascape. The image shows the histogram of the top 20 enriched pathway. **H** The Venn diagrams were used to screen the differential expression related with fatty acid metabolism in LSCs. **I** Univariate COX regression analysis of the 9 potential genes in TCGA-LAML. **J** Consensus matrix when k = 4. **K** Kaplan-Meier OS curves for AML patients among C1, C2, C3, and C4 in TCGA-LAML. This table under Kaplan-Meier OS curves shows that the remaining patients who do not have the end point event (death) under the indicated time in this subgroup. They are at risk of an endpoint event, which is called number at risk. **L** The expression of LFMGs among C1, C2, C3, and C4. **M** Heatmap of correlation between lipid metabolism-associated genes in LSCs with clinicopathological characteristics of AML patients in the TCGA-LAML cohort. **N** Lasso COX regression analysis of five OS-related genes. **O** The overall survival (OS) in the TCGA-LAML cohort database was analyzed by the univariate COX regression with the 5 potential genes and summarized in Forest plots. *p < 0.05; **p < 0.01; ***p < 0.001; ****p < 0.0001; ns, not significant.

To explore whether lipid metabolism is related to the progress and prognosis of AML, we first performed the survival analyses of four fatty acid metabolism-related gene sets (KEGG, Hallmark, Reactom and WikiPathway) quantified by single sample gene set enrichment analysis (ssGSEA), and found that the prognosis of AML patients with high fatty acid metabolism is worse (Fig. 1A–D). Afterwards, all the genes related to fatty acid metabolism in the four fatty acid metabolism-related gene sets were collected as shown in Fig. S1A. As LSCs control the recurrence and refractory of AML patients, the fatty acid metabolism-related genes which highly expressed in LSCs may be a potential therapeutic target. Thus, we urge to seek the differentially expressed genes between LSCs and HSCs, and the three datasets of GSE68172 (5 HSC samples and 19 LSC samples), GSE17054 (4 HSC samples and 9 LSC samples), and GSE24395 (5 HSC samples and 12 LSC samples) were utilized to dig up the potential effectors by robust rank aggregation method. (Fig. S1B). As shown, a total of 211 differentially expressed genes in LSCs were enriched, including 109 up-regulated genes and 102 down-regulated genes (Fig. 1E). Consistently, we performed function analysis to investigate the biological processes and the corresponding pathways of differentially expressed genes in LSCs by Metascape, and found some lipid metabolism-related biological processes, such as “glycerophospholipid metabolic process” in up-regulated differentially expressed genes (Fig. 1F) and “lipid homeostasis process” in down-regulated differentially expressed genes (Fig. 1G). Thus, we screened the potential regulators of fatty acid metabolism in LSCs by intersecting the differentially expressed genes in LSCs (Fig. 1E) with the genes related to fatty acid metabolism (Fig. S1A), and 9 potential genes were founded (Fig. 1H). The prognostic significance of 9 potential genes in TCGA-LAML were analyzed by univariate COX regression, and six genes (LGALS1, ALDH1A1, AADAT, ELOVL7, ACOX2, and ACSM3) were significantly correlated with the prognosis of AML patients. LGALS1, ALDH1A1, AADAT, ELOVL7, and ACOX2 were the risk factors for the prognosis of AML patients, while ACSM3 was the protective factor (Fig. 1I).

Next, we performed the consensus clustering analysis using R package “ConsensusClusterPlus” with cluster variable range sited from 2 to 10 based on these six potential effectors to identify the potential fatty acid metabolism-related subtypes in AML patients. First, we performed cumulative distribution function (CDF) plots, and found that when k = 4, the descending slope of CDF is smaller compared with k = 2, k = 3, and k = 5, and CDF reached an approximate maximum (Fig. S1C). Also, delta area plot was made. As shown in Fig. S1D, when k = 5, the change of area under curve was slighter than k = 4. In brief, 4 clusters are suitable for the highest intragroup correlations and the lowest intergroup correlations, which can be observed in Fig. S1E. These data indicated that k = 4 showed distinguished clustering stability with the highest intragroup correlations and the lowest intergroup correlations. Thus, we acquired four clusters (C1, C2, C3, and C4) (Fig. 1J). In addition, the clusters were validated by PCA analysis (Fig. S1F). Furthermore, we analyzed the survival of C1, C2, C3, and C4 clusters, and found that C1 had the worst prognosis and C4 had the best prognosis (C1 < C3 < C2 < C4) (Fig. 1K). Together, the relative expression of the six potential effectors were detected, and found most risk factors (LGALS1, ALDH1A1, ELOVL7 and ACOX2) were high expressed in C1 and C3 clusters, and the protective factor (ACSM3) were high expressed in C2 and C4 clusters (Fig. 1L). Next, we investigated the association of LSCs and fatty acid metabolism-related clusters with classical clinical features of AML in the TCGA-LAML cohort, LSCs and fatty acid metabolism related-clusters, survival status, overall survival, age, sex, FAB typing, WAB number, cytogenetic risk, CD34, proportion of progenitor cells, proportion of precursor cells in bone marrow, FLT3 gene mutation, NPM1 mutation, and expression profile of each gene were used as annotations (Fig. 1M). The results showed that the survival status was poorer (Fig. S1G), the percentage in M5 (the worst prognosis subtype) [24] (Fig. S1H), WBC counts (Fig. S1I), and poor cytogenetics risk (Fig. S1J) were higher in C1 and C3 clusters (worst prognosis) compared with C2 and C4 clusters, which indicated that the four clusters were successfully identified based on the potential six genes of AML.

To better assist clinicians in accurately predicting the prognosis of AML patients, we try to construct the LSCs and fatty acid metabolism-related risk score (LFMRS) based on the six potential genes. The Least Absolute Shrinkage and Selection Operator (LASSO) regression algorithm determined five OS-related genes based on the optimum λ value and the minimum partial likelihood of deviance (Fig. 1N, S1K). LASSO coefficients of the five potential genes showed that LGALS1 (0.260), ELOVL7 (0.215), ALDH1A1 (0.045), and ACOX2 (0.013) were risk factors and ACSM3 (-0.138) was a protective factor (Fig. 1O).

### Verification of prognostic model (LFMRS) in AML

**Fig. 2:**
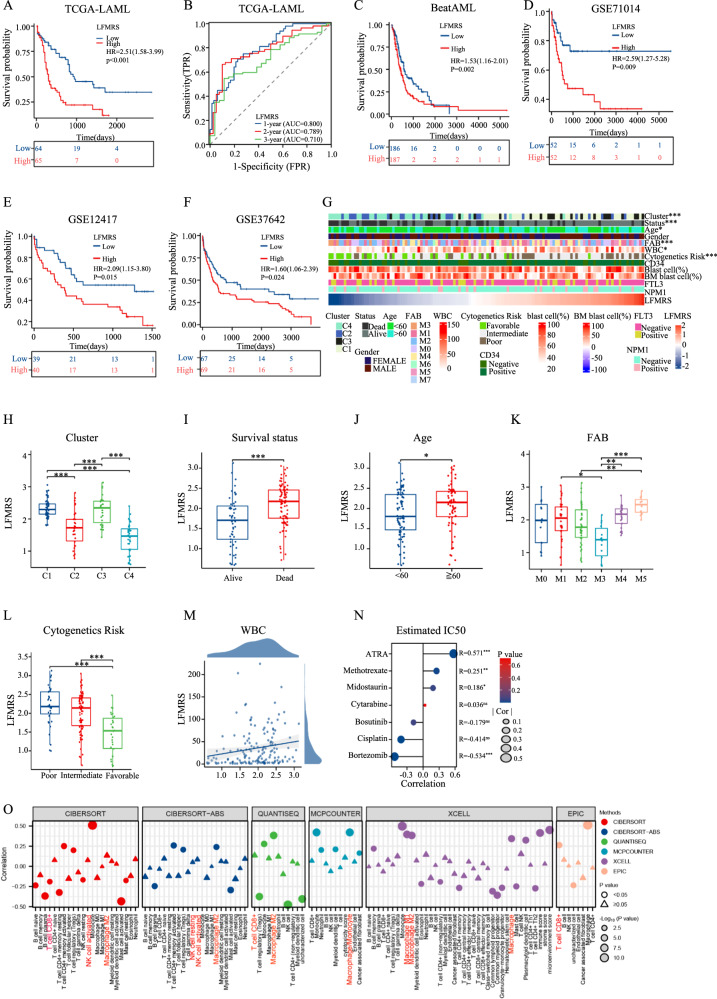
Verification of prognostic model (LFMRS) in AML. **A** Kaplan-Meier analysis of OS between high- and low-LFMRS groups in the TCGA cohort. **B** The 1-, 2- and 3-year ROC curves of the LFMRS in the TCGA cohort. Kaplan-Meier analyses of OS between high- and low-LFMRS groups in the BeatAML database (**C**), GSE71014 (**D**), GSE12417 (**E**) and GSE37642 (**F**) cohorts. **G** Overview of the correspondence between LFMRS and other clinical features of AML patients. LFMRS expression among distinct clusters (**H**), between alive and dead patients (**I**), between <60 and ≥60 patients (**J**), among distinct FAB subtypes (**K**), among different cytogenetic risks (**L**) of AML. **M** The correlation of LFMRS with WBC counts. **N** The correlation of LFMRS with IC50 values of first-line drugs from GDSC database quantified by pRRophetic. **O** Correlation of LFMRS with immune cells quantified by CIBERSORT, CIBERSORT-ABS, QUANTISEQ, MCPCOUNTER, XCELL and EPIC in AML. *p < 0.05; **p < 0.01; ***p < 0.001; ****p < 0.0001; ns not significant.

According to the median risk score of LFMRS in training cohort of TCGA, AML patients were divided into low-risk group and high-risk group (Fig. S2A). Not surprisingly, patients in high-risk group had shorter survival time and worse prognosis than those in low-risk group (Fig. 2A). Moreover, the sensitivity and specificity of LFMRS were estimated through time-dependent receiver operating characteristic (ROC) analysis, and the areas under the curve (AUCs) for one-year, two-year, and three-year overall survival were 0.800, 0.789, and 0.710, respectively with significant *p* values (Fig. 2B). For verifying the reliability of LFMRS, the same analysis is carried out in the four databases of BeatAML (Fig. 2C, S2B), GSE71014 (Fig. 2D, S2C), GSE12417 (Fig. 2E, S2D), and GSE37642 (Fig. 2F, S2E), and the results show that the constructed LFMRS has a high prognostic reliability.

Next, we stratified the AML patients into high-risk and low-risk groups according to their LFMRS scores and assessed their clinical parameters, and found that the distribution of the clusters, survival status, age, FAB typing, WBC counts, and cytogenetic risk-based risk groups were different between the LFMRS high- and low-risk groups, while other clinical features showed no significance (Fig. 2G). We also analyzed the LFMRS risk values among the clusters, survival status, age, FAB classification, WBC counts, and cytogenetic risk, and found that LFMRS were higher in patients with C1 and C3 clusters (Fig. 2H), poor survival status (Fig. 2I), older than 60 years old (Fig. 2J), M5 type in FAB classification (Fig. 2K), and poor cytogenetic risk (Fig. 2L). LFMRS risk values was positively correlated with WBC counts (Fig. 2M). Moreover, we detected the correlation between LFMRS and estimated drug IC50 quantified by pRRophetic method (Fig. S2F), and found that LFMRS was the most sensitive to parthenolide and least sensitive to ATRA in AML (Fig. S2G). The same result was also found through the analysis of the correlation between clinical applied drugs in AML and LFMRS, which indicating that LFMRS may be the key factor of drug resistance, especially in retinoic acid treatment of AML (Fig. 2N). Meanwhile, we also assessed the correlation between LFMRS and immune cells using CIBERSORT, CIBERSORT-ABS, QUANTISEQ, MCPCOUNTER, XCELL, and EPIC algorithms, and found that macrophage, CD8^+^ T cells, NK cell, et. al. were closely related to LFMRS (Fig. 2O). Thus, these data indicated that LFMRS risk classification were consistent with current risk factors.

To determine whether the LFMRS is independently correlated with the OS of AML patients, we first performed univariate COX regression analysis. Through analyzing the prognostic value of LFMRS together with other common prognostic factors (age, gender, WBC counter, cytogenetics risk, CD34 expression, the percentage of blast cell and BM blast cell, FLT3 mutation, and NPM1 mutation), we found that age, cytogenetic risk and LFMRS were associated with prognosis of patients with AML (Fig. S3A). Furthermore, we also performed multivariate COX regression analysis, and the results also showed that age and LFMRS were independent prognostic factors (Fig. S3B). Next, we established a prognostic nomogram integrating age and LFMRS (Fig. S3C), and the calibration curve of nomogram showed high concordance between the predicted and actual probabilities of 1-, 2- and 3-year survival (Fig. S3D). The reliability of the predictive effect of the nomogram on prognosis were further verified in 1-, 2-, and 3-year prognosis by ROC curves (Fig. S3E). Overall, these results indicated that LFMRS is an independent risk factor, can be combined with age for better precisely predicting the survival time of AML patients.

### LGALS1 is highly expressed in LSCs and associated with poor prognosis of AML patients

**Fig. 3:**
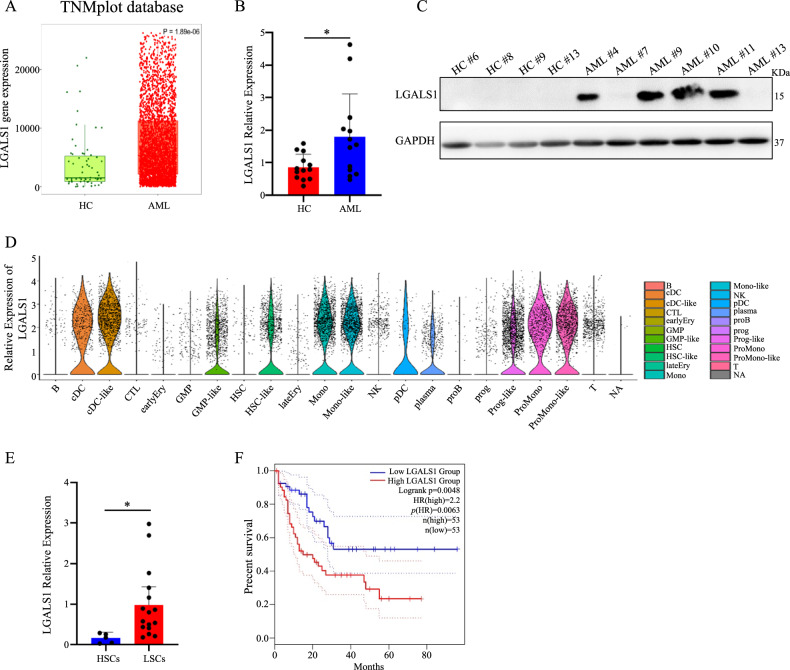
LGALS1 is highly expressed in LSCs and associated with poor prognosis of AML patients. **A** The transcript levels of LGALS1 in AML samples compared with that in healthy individuals were identified from TNMplot database. **B** The mRNA levels of LGALS1 in primary AML cases (*n* = 13, AML#1-AML#13) and healthy control cases (*n* = 13). **C** The protein levels of LGALS1 in indicated primary AML cases and healthy control cases. **D** The relative mRNA expression of LAGLS1 from the single cell sequencing data of GSE116256. **E** The mRNA levels of LGALS1 in HSCs and LSCs from healthy control cases and AML cases, respectively (LSCs from AML#1-AML#16). **F** Kaplan-Meier plots of overall survival in TCGA cohorts for AML patients, stratified on the basis of LGALS1 expression above (LGALS1^high^) or below (LGALS1^low^) the median. *p < 0.05; **p < 0.01; ***p < 0.001; ****p < 0.0001; ns, not significant.

LGALS1 is the only member of LFMRS model which is highly expressed in LSCs compared with HSCs (Fig. 1E), has the most significant prognostic effect with the highest HR and the lowest *p* value (Fig. 1I). and is the molecule with the highest prognosis risk (Fig. 1O). Additionally, LGALS1 is highly expressed in worst-prognosis cluster C1 in AML (Fig. 1L) and is positively correlated with LFMRS score (Fig. S4A). Thus, LGALS1 caught our attention to further explore the function and mechanism AML to verify LFMRS model. To validated whether LGALS1 is highly expressed in LSCs, we first analyzed LGALS1 expression through TNMplot database (Fig. 3A) and TARGET database (Fig. S4B), and found that LGALS1 was high expressed in AML patients than healthy individuals. Meanwhile, we collected bone marrow samples from AML patients, also found that LGALS1 was aberrantly overexpressed in AML samples relative to healthy controls at both the mRNA level (Fig. 3B) and protein level (Fig. 3C). Next, we analyzed the single cell sequencing data from GSE116256, and found that LGALS1 expression was higher in LSC-like (HSC-like and GMP-like) compared with HSC-like (HSC and GMP) (Fig. 3D). Also, we sorted LSCs from AML patients and HSCs in individual of healthy, and found that LGALS1 expression was higher in LSCs than HSCs (Fig. 3E). Moreover, the elevated expression of LGALS1 correlated with poor survival of AML patients (Fig. 3F). Thus, LGALS1 is highly expressed in LSCs and associated with poor prognosis of AML patients.

In addition, we also assessed the expression and prognosis of others members of LFMRS model. As shown in Fig. S4C, ACSM3 was high expressed in AML than health individual, while ELOVL7 and ALDH1A1, and ACOX2 were low expressed through TARGET database. Moreover, there were no significant difference of ACSM3 and ACOX2 expressions between AML samples relative to healthy controls, while ELOVL7 and ALDH1A1 were aberrantly low-expressed in our collecting samples (Fig. S4D). We also revealed that ELOVL7 and ACOX2 were lower in LSCs compared with HSCs, and there were no significant difference of ALDH1A1 and ACSM3 expressions between two groups (Fig. S4E). The high expression of ELOVL7, ALDH1A1, and ACOX2 correlated with poor survival of AML patients, while high expression of ACSM3 related with good survival of AML patients (Fig. S4F). These results are mostly consistent with LFMRS model.

### LGALS1 promotes cell proliferation and inhibits cell apoptosis

**Fig. 4:**
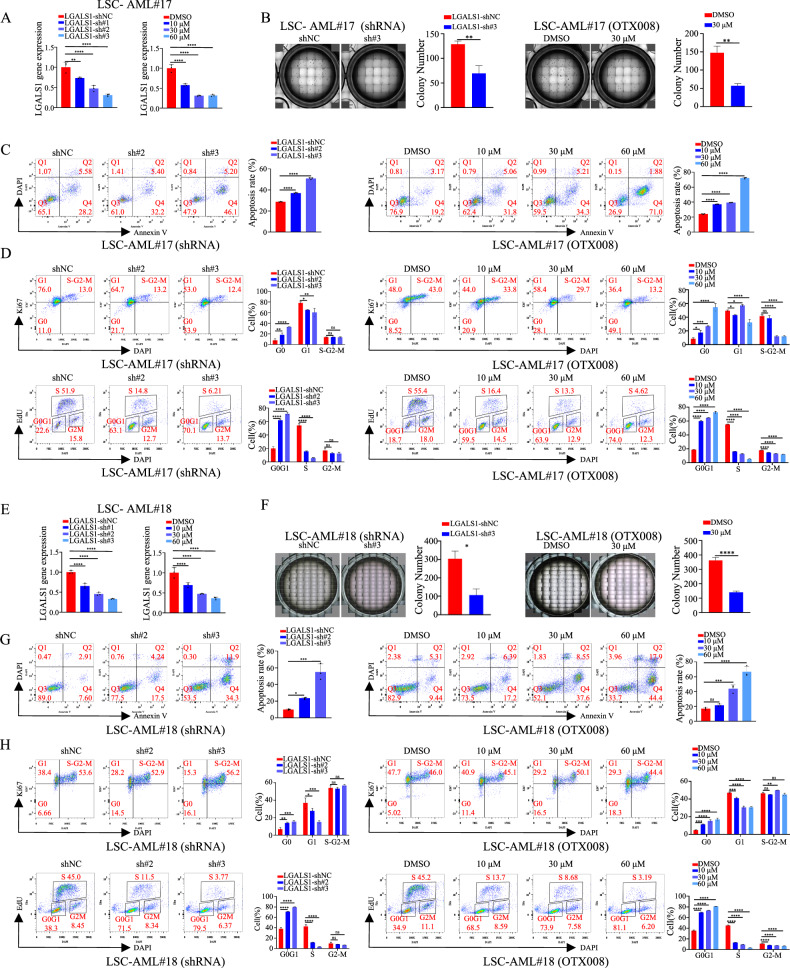
LGALS1 promotes cell proliferation and inhibits cell apoptosis of LSCs. **A**–**D** LSCs were sorted from AML#17. **A** Efficiencies of LGALS1 silence in LSCs were determined by qRT-PCR. **B** Cell growth was determined by colony formation assay under a light microscope, and the percentage of colony formation units were shown. **C** Cell apoptosis was determined by flow cytometric analysis. **D** Cell cycle distribution was detected by flow cytometric analysis via Ki67 staining (upper) and EdU staining (lower), respectively, and the bar graph showed the percentage of G0/G1, S, and G2/M phase cells. **E**–**H** LSCs were sorted from AML#18. **E** Efficiencies of LGALS1 silence in LSCs were determined by qRT-PCR. **F** Cell growth was determined by colony formation assay under a light microscope, and the percentage of colony formation units were shown. **G** Cell apoptosis was determined by flow cytometric analysis. **H** Cell cycle distribution was detected by flow cytometric analysis via Ki67 staining (upper) and EdU staining (lower), respectively, and the bar graph showed the percentage of G0/G1, S, and G2/M phase cells. *p < 0.05; **p < 0.01; ***p < 0.001; ****p < 0.0001; ns not significant.

**Fig. 5:**
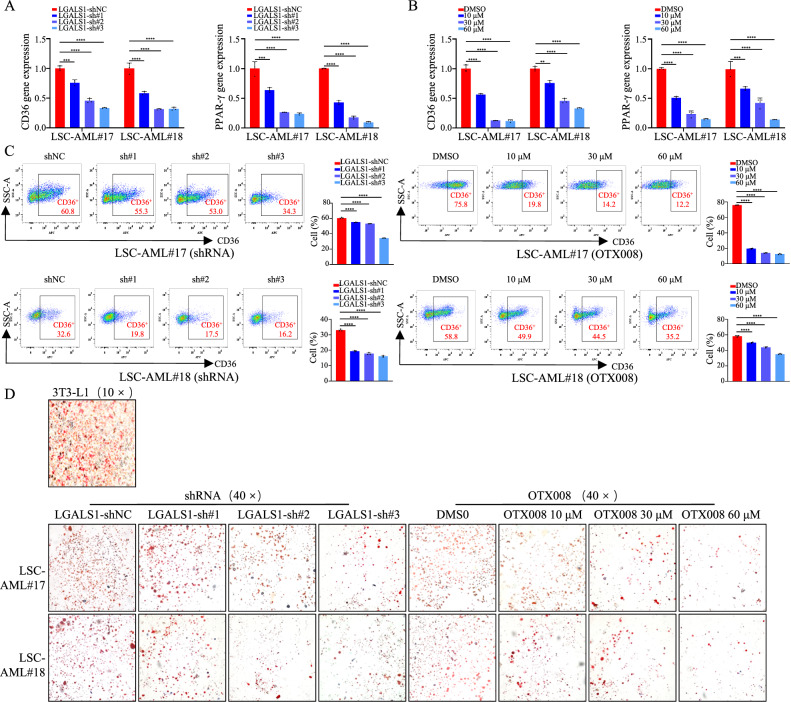
LGALS1 plays a key role in lipid metabolism reprogramming of LSCs. **A**–**D** LSCs transfected with shRNA against GALS1, or treated with DMSO or OTX008 were cultured. **A**, **B** The mRNA levels of CD36 and PPAR-γ were detected by qRT-PCR. **C** The protein levels of CD36 were determined by FCM using anti-CD36-APC (1:100, BioLegend, America). **D** Representative images of Oil Red O staining. Differentiated 3T3-L1 cell was used as a positive control. *p < 0.05; **p < 0.01; ***p < 0.001; ****p < 0.0001; ns not significant.

To explore the function of LGALS1 in LSCs, we sorted LSCs (CD34^+^CD38^−^) from two AML patients. As shown in Fig. 4A–H, depletion of LGALS1 expression by shRNA (Figs. 4A, E) or inhibiting LGALS1 expression (Figs. 4A, E) using OTX008 (a specific inhibitor of LGALS1) [25] impaired cell proliferation *via* colony formation assay (Figs. 4B, F), enhanced cell apoptosis *via* flow cytometric analysis (Figs. 4C, G), and led to a decrease in the fraction of cells in S phase (presented the rate of proliferation) and increase in that in G0 phase (Figs. 4D, H). We also determined LGALS1 expression in a set of leukemia cells, and found a relative stronger endogenous LGALS1 was seen in HEL, THP1, MV411, and NB4 cells (Fig. S5A, S5B). Therefore, we explored the function in AML using HEL and THP1 cells, and found that suppression of LGALS1 (Fig. S5C, S6A) in THP1 and HEL cells also impaired cell proliferation (Fig. S5D, S6B), enhanced cell apoptosis (Fig. S5E, S6C), and led to a decrease in the fraction of cells in S phase (Fig. 5F, S6D). Thus, LGALS1 promotes cell proliferation and inhibits cell apoptosis in LSCs and leukemia cells in vitro.

### LGALS1 contributes to lipid metabolism reprogramming

Lipid metabolism is regulated by a combination of the uptake and export of fatty acids, de novo lipogenesis, and fat utilization by β-oxidation [26]. It is reported that LGALS1 is associated with lipid synthesis in adipocyte by activation of peroxisome proliferator-activated receptor gamma (PPARγ) in adipose cell [26]. Galectin-1 has also been proposed to regulate adipogenesis and adipose inflammation by binding to CD146 [27]. While, whether LGALS1 regulates lipid metabolism in leukemia cells remains unknown. Therefore, we refine the effect of LGALS1 on the lipid uptake and de novo lipogenesis in LSCs and leukemia cells. First, we detected the expression of gene-related to lipid uptake (CD36) and de novo lipogenesis (PPAR-γ, FASN, ACC), and found that depletion of LGALS1 expression by shRNA or inhibiting LGALS1 expression using OTX008 decreased CD36 and PPAR-γ expression (Fig. 5A–C, S7A–S7C), which indicated that LGALS1 may enhance lipid accumulation in LSCs, HEL, and THP1 cells. Not surprisingly, Oil Red O staining showed that restrained LGALS1 significantly reduced the accumulation of lipid droplets in LSCs and leukemia cells (Fig. 5D, S7D). The above data indicate that LGALS1 enhances the accumulation of fatty acids in LSCs and leukemia cells.

### LGALS1 enhances lipid metabolism reprogramming, an immunosuppressive microenvironment, and AML progression in vivo

**Fig. 6:**
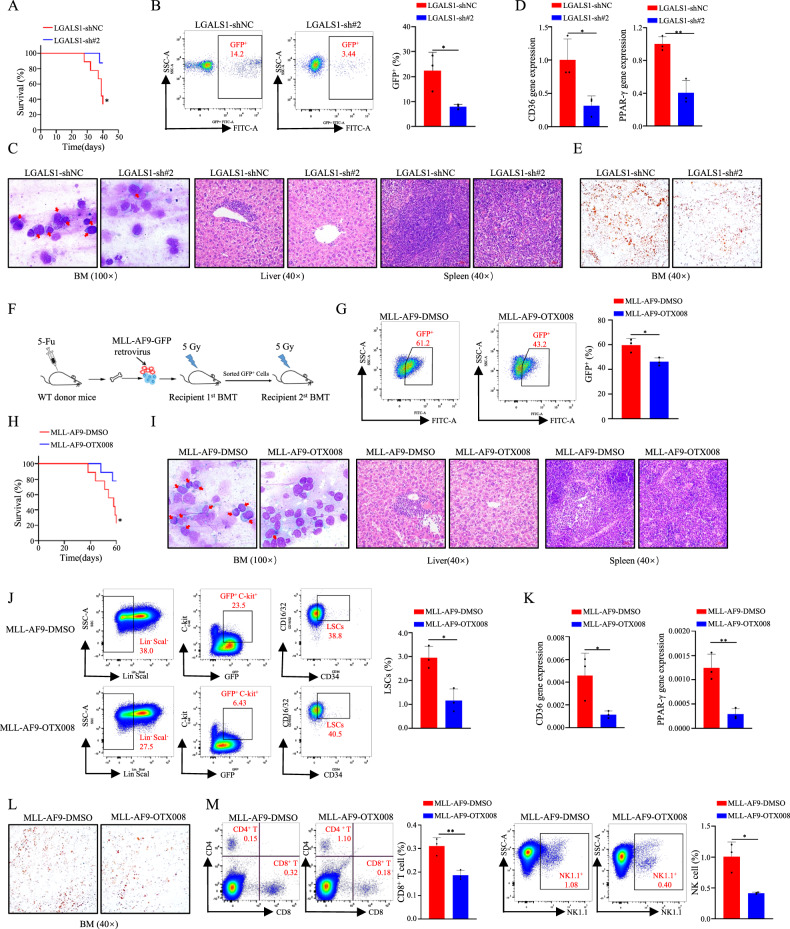
LGALS1 enhances lipid metabolism reprogramming, an immunosuppressive microenvironment, and AML progression in vivo. **A** Kaplan-Meier analysis of the survival curves of the mice in each group (*n* = 5). **B** The percentage of GFP^+^ leukemia cell in bone marrow were detected through flow cytometric analysis (*n* = 3). **C** Immature cells from the bone marrow were checked using Wright’s stain (Left), and spleen and liver infiltration were analyzed by H&E staining (Right). The representative pictures were shown. **D** The relative expression of CD36 and PPAR-γ in GFP^+^ leukemia cells were determined by qRT-PCR (*n* = 3). **E** Representative images of Oil Red O staining. **F** The schema chart of MLL-AF9-induced leukemia was shown (*n* = 3). **G** The percentage of GFP^+^ leukemia cell in peripheral blood were detected through flow cytometric analysis (*n* = 3). **H** Kaplan-Meier analysis of the survival curves of the mice in each group (*n* = 7). **I** Immature cells from the bone marrow were checked using Wright’s stain (Left), and spleen and liver infiltration were analyzed by H&E staining (Right). The representative pictures were shown. **J** The proportion of LSCs in bone marrow was detected by flow cytometric analysis (*n* = 3). **K** The relative expression of CD36 and PPAR-γ in GFP^+^ leukemia cells were determined by qRT-PCR (*n* = 3). **L** Representative images of Oil Red O staining. **M** The proportion of CD8^+^ T cells and NK cells in bone marrow were determined by flow cytometric analysis (*n* = 3). *p < 0.05; **p < 0.01; ***p < 0.001; ****p < 0.0001; ns, not significant.

To examine the mitogenic effect of LGALS1 in vivo, engineered HEL cells were injected into NOD/SCID mice, as expected, we found that the mice injected with shLGALS1 cells showed longer survival time than those injected with shNC cells (Fig. 6A). LGALS1 knockdown inhibited leukemogenesis (Fig. 6B). Next, leukemia cell infiltration was assessed. As shown in Fig. 6C, LGALS1 knockdown reduced leukemic cells in the bone marrow, and curbed liver and splenic infiltration as detected by H&E staining (Fig. 6C). Not surprisingly, the levels of CD36, PPAR-γ, and lipid droplet were reduced in xenografts from mice receiving LGALS1 knockdown cells (Fig. 6D, E). These results suggest that LGALS1 regulates expansion and lipid metabolism reprogramming of leukemic cells.

As LFMRS was closely related to the immunosuppressive state of AML (Fig. 2O), we further explore whether LGALS1 regulates the immune effect of immune cells (macrophage, CD8^+^ T cells, and NK cells) in vivo by using the MLL-AF9 retroviral transduction system (Fig. 6F). LSK cells transduced with MLL-AF9 virus from wildtype C57/B6L mice, then injected into unirradiated mice via tail vein. As shown in Fig. 6G–L, treatment with OTX008 inhibited leukemogenesis (Fig. 6G), promoted survival time (Fig. 6H), reduced leukemic cells in the bone marrow (Fig. 6I), curbed liver and splenic infiltration (Fig. 6I), and decreased LSC frequency in the bone marrow *via* flow analysis (Fig. 6J), and reduced CD36, PPAR-γ, and lipid droplet levels (Fig. 6K, L). Meanwhile, we detected the immune cell counts, and found that treatment with OTX008 lead to a decrease in the counts of CD8^+^ T cells, and NK cells (Fig. 6M). In general, LGALS1 enhances lipid metabolism reprogramming, an immunosuppressive microenvironment, and AML progression in vivo.

## Discussion

Increasing evidences have indicated that the remolding of tumor lipid metabolism could lead to tumor progression and local immunosuppression in the tumor microenvironment [28]. Due to the limited efficacy of diverse therapeutic methods of AML, like chemotherapy and hematopoietic stem cell transplantation, exploring novel biomarkers is urgently needed, which could not only predict the OS of AML patients, but also be utilized to guide anti-leukemia therapy. Consequently, recent studies have shed more light on the aberrant lipid metabolism and its effect on the immune microenvironment in the context of AML.

In this work, we successfully identified five lipid metabolism-related genes which were associated with AML prognosis, including LGALS1, ELOVL7, ALDH1A1, ACOX2, and ACSM3, and construct LFMRS model *via* a series of bioinformatics analysis. Notably, this LFMRS model can discriminate high-risk from low-risk population, and the patients in low-risk group were proved to have longer OS than those in high-risk groups in five databases (TCGA, BeatAML, GSE71014, GSE12417, and GSE37642). Additionally, LFMRS model can potentially estimate the different prognosis for low- and high- groups based on clinical parameters, such as clusters, survival status, age, FAB typing, WBC counts, cytogenetic risk-based risk groups. We also found that LFMRS can assess drug resistance and immune function in microenvironment. Furthermore, LFMRS can be an independent risk factor and predict the survival time of AML patients combined with age through univariate COX regression analysis and multivariate COX regression analysis. Consequently, the lipid metabolism-related signature identified may be involved in the occurrence and development of AML, rendering its potential as the valuable clinical biomarker.

LGALS1, a beta-galactoside-binding protein, is associated with lipid metabolism, such as enhancing adipogenesis and adipose inflammation [27]. Recently, the carcinogenesis of LGALS1 has been gradually revealed, such as in non-small cell lung cancer cells [29], head and neck cancer [30]. Peter P, et. al found LGALS1 acted as a pro-survival molecule in AML [31]. Kening et al. revealed that LGALS1 was upregulated in refractory AML patients and its inhibition could enhance the chemotherapy in AML patients [25]. While, the enhancement of LGALS1 on AML progression lacks of sufficient evidence, and whether LGALS1 regulates lipid metabolism in leukemia remains unknown. Here, we first found that LGALS1 was highly expressed in LSCs and associated with poor prognosis of AML patients *via* analyzing RNA-seq data in BeatAML database, single cell sequencing data in TCGA database, and detecting the expression in collected AML samples. LGALS1 promoted cell proliferation and inhibited cell apoptosis *via* shRNA of LGALS1 and a specific inhibitor of LGALS1 in leukemia cells (HEL and THP1) and LSCs in vitro, and enhanced AML progression in vivo. By detecting the genes related to fatty acid metabolism, we discovered that LGALS1 enhanced CD36 (related with lipid uptake) and PPAR-γ (related with de novo lipogenesis) expression, and lipid accumulation, which was consistent with the discovery that lipid plays vital role in AML survival. Matthew et. al, reported that very long chain fatty acid metabolism is required in acute myeloid leukemia [32]. Fatty acid oxidation upregulation has importance role in ven/aza resistance of LSCs [33].

LGALS1 acts as the immune heterogeneity and immunosuppression in plenty of cancer, such as glioblastoma [34], clear cell renal carcinoma [35], non-small cell lung cancer [36]. Here, we first revealed that treatment with OTX008 (a specific inhibitor of LGALS1) lead to a decrease in the counts of CD8^+^ T cells and NK cells. As reported that lipid is vital to T cell and macrophage function [37, 38], also tumor immune escape [39]. As leukemia cells absorb lipid from microenvironment through LGALS1, which may lead to less uptake of fatty acids for immune cells to maintain their function. However, Currently, we still cannot address how LGALS1 regulated CD8^+^ T cells and NK cells, and thus further exploration is warranted.

The members of LFMRS may coordinate with each other to regulate lipid metabolism and AML progress. ELOVL7, as a lipogenic gene, enhances fatty acid synthesis *via* coding a long-chain fatty acid elongase [40]. It was reported that ELOVL7 was involved in prostate cancer growth [40, 41]. While, the specific mechanism of ELOVL7 on fatty acid metabolism in AML is not revealed. ALDH1A1, aldehyde dehydrogenase 1A1, is the rate-limiting enzymes that convert retinaldehyde to retinoic acid, and ALDH1A1 deficiency significantly attenuated triacylglycerol synthesis [42]. Moreover, ALDH1A1 is a marker of cancer stem cells, and is involved in LSC property maintenance [43]. ACOX2, as a β-oxidation gene, participated in lipid degradation [44], and could be used as both tumor suppressor gene and tumor promoter gene [45, 46]. The function of ACOX2 in leukemia needs further exploration. ACSM3, acyl-CoA synthetase medium-chain family member 3, perform the initial reaction for fatty acid metabolism by trapping fatty acid within a cell and activating it for metabolism [47]. Decreased ACSM3 expression is indicative of deregulated fatty acid oxidation and a poor survival in liver cancer [48]. Moreover, ACSM3 could repress the cell proliferative activity and facilitated induction of apoptosis and cell cycle arrest in AML cells [49]. Here, we found that high expression of ELOVL7, ALDH1A1, and ACOX2 were correlated with poor survival of AML patients (Fig. S4F). While, ACSM3 was a protective factor for AML (Fig. S4F). Moreover, we also performed the correlation analysis of LGALS1 and other members of LFMRS, and revealed that LGALS1 is positively correlated with ACOX2 expression, and negatively correlated with ELOVL7 and ACSM3 (Fig. S8A, S8C-S8D). While, there is no significant difference between LGALS1 and ALDH1A1 (Fig. S8B). LGALS1 and other members of LFMRS model may regulate the homeostasis of lipid metabolism and the progress of leukemia cells.

We performed a systematic analysis of the regulatory functions of LFMRS in AML and their effect on prognosis, and revealed that LFMRS as independent prognostic factors for AML. Furthermore, we explore the function of LGALS1 in AML in vitro and in vivo. We found that LGALS1 was highly expressed in LSCs and associated with poor prognosis of AML patients, and regulated AML progression, lipid metabolism reprogramming, and immune cell counts. Our findings may provide a new prediction and therapeutic target to AML.

### Supplementary information

Supplementary Figure

sup figure legends

CDDIS-24-0762RR WB daw data

## Data Availability

All data generated or analyzed during this study are included in this article and its supplementary files.
