# Supplementary material for: A novel LGALS1-depended and immune-associated fatty acid metabolism risk model in acute myeloid leukemia stem cells
Source: Cell Death Dis. 2024 Jul 5;15(7):482. doi: 10.1038/s41419-024-06865-6 (PMC11224233; doi:10.1038/s41419-024-06865-6)
Supplement: Supplementary file 1 — Supplementary Figure [file 41419_2024_6865_MOESM1_ESM.pptx]

## Slide 1
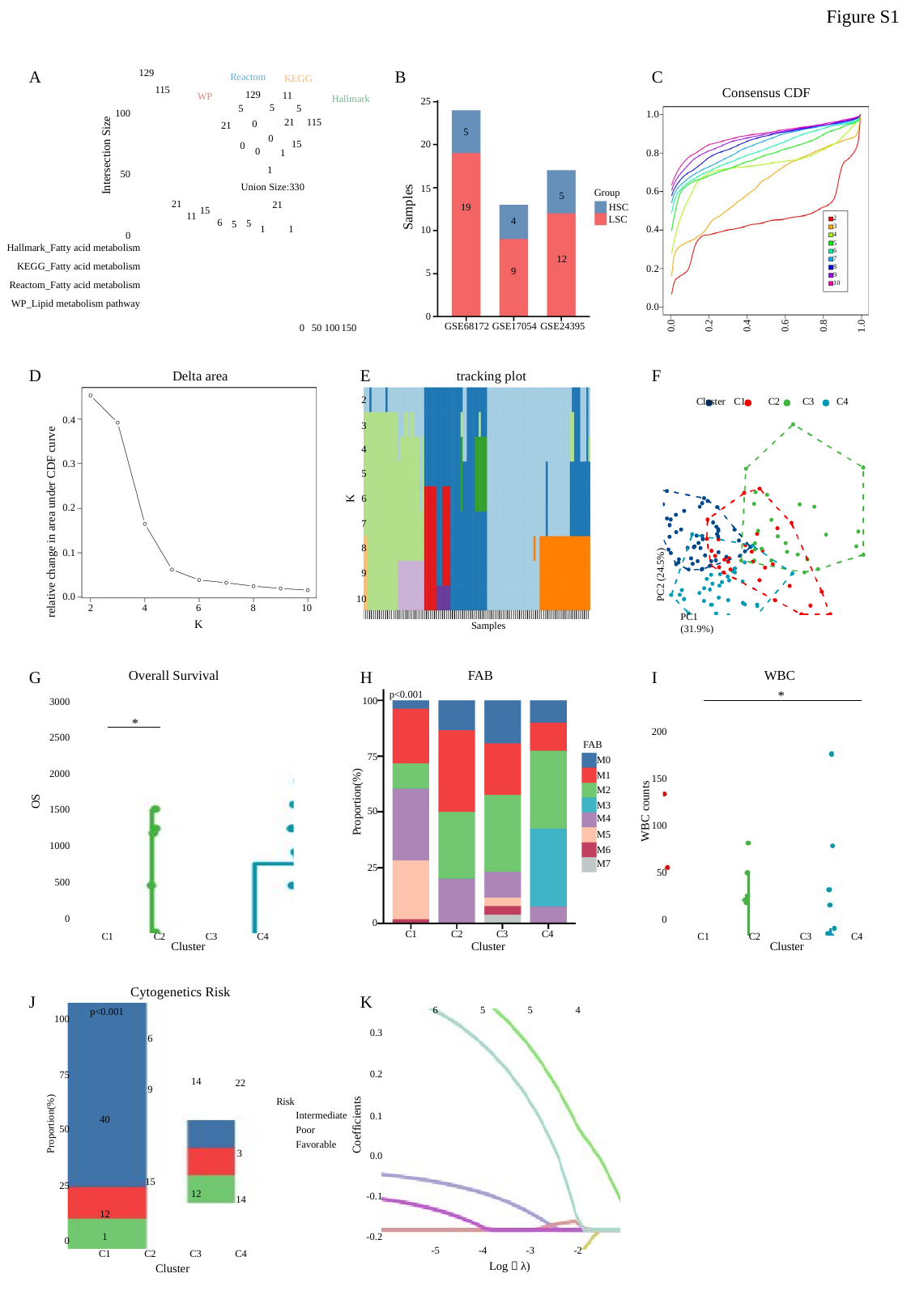

Figure S1
A
B
C
129
Reactom
KEGG
115
129
11
WP
Hallmark
5
5
5
100
115
21
0
21
0
15
0
0
1
Intersection Size
1
50
21
21
15
11
6
5
5
1
1
0
Hallmark_Fatty acid metabolism
KEGG_Fatty acid metabolism
Reactom_Fatty acid metabolism
WP_Lipid metabolism pathway
0
50
100
150
Consensus CDF
25
5
20
15
5
19
HSC
4
10
12
9
5
0
GSE68172
GSE17054
GSE24395
LSC
Group
Samples
1.0
0.8
Union Size:330
0.6
2
3
0.4
4
5
6
7
8
0.2
9
10
0.0
0.0
0.2
0.4
0.6
0.8
1.0
D
E
F
Delta area
tracking plot
2
Cluster
C1
C2
C3
C4
PC2 (24.5%)
PC1 (31.9%)
0.4
3
4
0.3
5
K
6
0.2
7
8
relative change in area under CDF curve
0.1
9
0.0
10
2
4
6
8
10
K
Samples
G
H
I
Overall Survival
FAB
p<0.001
100
FAB
75
M0
M1
M2
Proportion(%)
M3
50
M4
M5
M6
M7
25
0
C1
C2
C3
C4
Cluster
WBC
*
3000
*
200
*
2500
2000
150
WBC counts
OS
1500
100
1000
50
500
0
0
C1
C2
C3
C4
C1
C2
C3
C4
Cluster
Cluster
Cytogenetics Risk
J
K
6
5
5
4
0.3
0.2
0.1
Coefficients
0.0
-0.1
-0.2
-5
-4
-3
-2
Log（λ)
p<0.001
100
6
75
14
22
9
Risk
Intermediate
40
Proportion(%)
50
Poor
Favorable
3
15
25
12
14
12
1
0
C1
C2
C3
C4
Cluster

## Slide 2
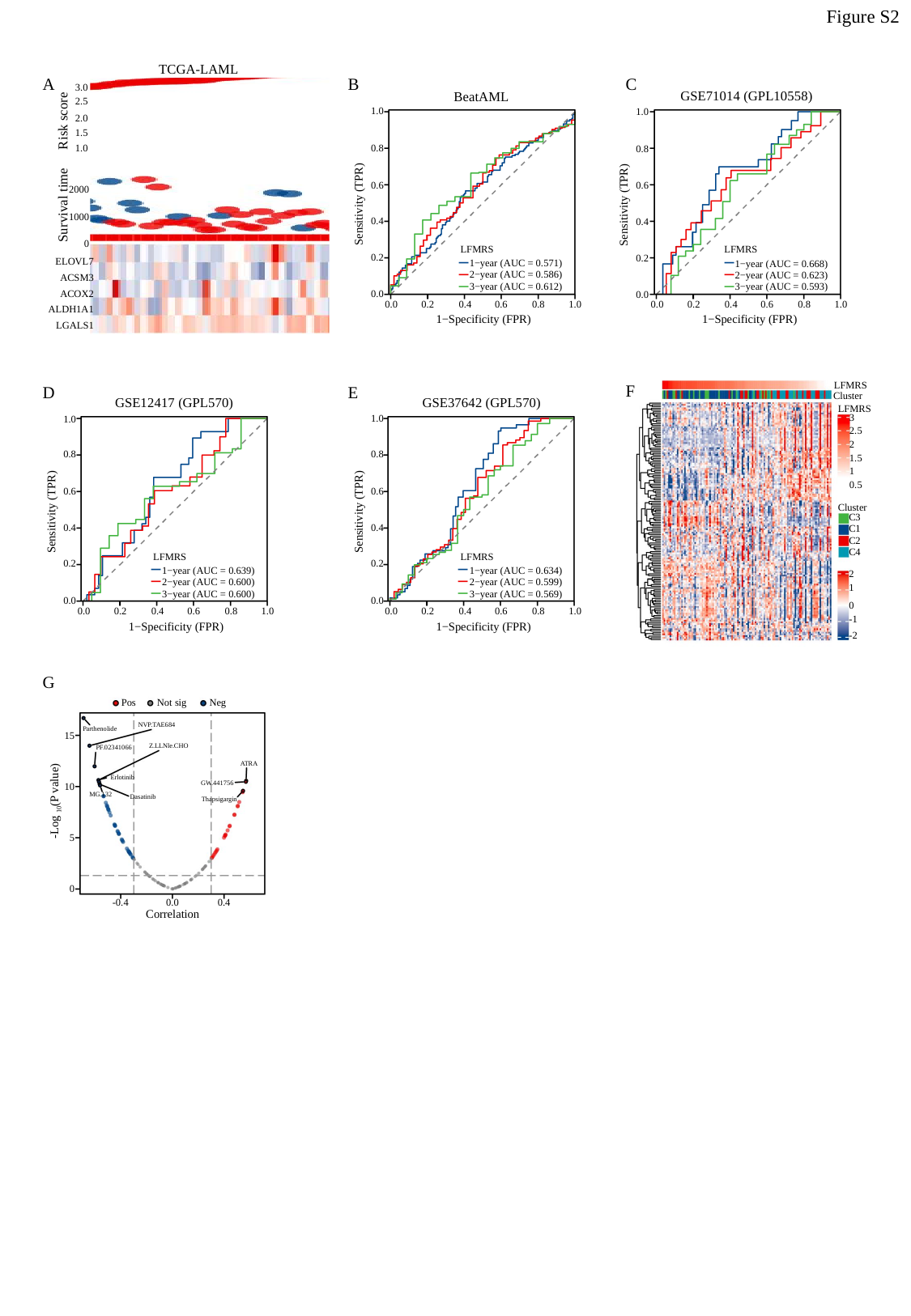

Figure S2
TCGA-LAML
3.0
2.5
2.0
Risk score
1.5
1.0
2000
Survival time
1000
0
ELOVL7
ACSM3
ACOX2
ALDH1A1
LGALS1
A
B
C
GSE71014 (GPL10558)
BeatAML
1.0
0.8
0.6
Sensitivity (TPR)
0.4
LFMRS
0.2
1−year (AUC = 0.571)
2−year (AUC = 0.586)
3−year (AUC = 0.612)
0.0
0.0
0.2
0.4
0.6
0.8
1.0
1−Specificity (FPR)
1.0
0.8
0.6
Sensitivity (TPR)
0.4
LFMRS
0.2
1−year (AUC = 0.668)
2−year (AUC = 0.623)
3−year (AUC = 0.593)
0.0
0.0
0.2
0.4
0.6
0.8
1.0
1−Specificity (FPR)
LFMRS
F
E
D
Cluster
GSE37642 (GPL570)
1.0
0.8
0.6
Sensitivity (TPR)
0.4
LFMRS
0.2
1−year (AUC = 0.634)
2−year (AUC = 0.599)
3−year (AUC = 0.569)
0.0
0.0
0.2
0.4
0.6
0.8
1.0
1−Specificity (FPR)
GSE12417 (GPL570)
1.0
0.8
0.6
Sensitivity (TPR)
0.4
LFMRS
0.2
1−year (AUC = 0.639)
2−year (AUC = 0.600)
3−year (AUC = 0.600)
0.0
0.0
0.2
0.4
0.6
0.8
1.0
1−Specificity (FPR)
LFMRS
3
2.5
2
1.5
1
0.5
Cluster
C3
C1
C2
C4
2
1
0
-1
-2
G
Pos
Not sig
Neg
NVP.TAE684
Parthenolide
15
Z.LLNle.CHO
PF.02341066
ATRA
Erlotinib
GW.441756
10
MG.132
Dasatinib
Thapsigargin
-Log 10(P value)
5
0
-0.4
0.0
0.4
Correlation

## Slide 3
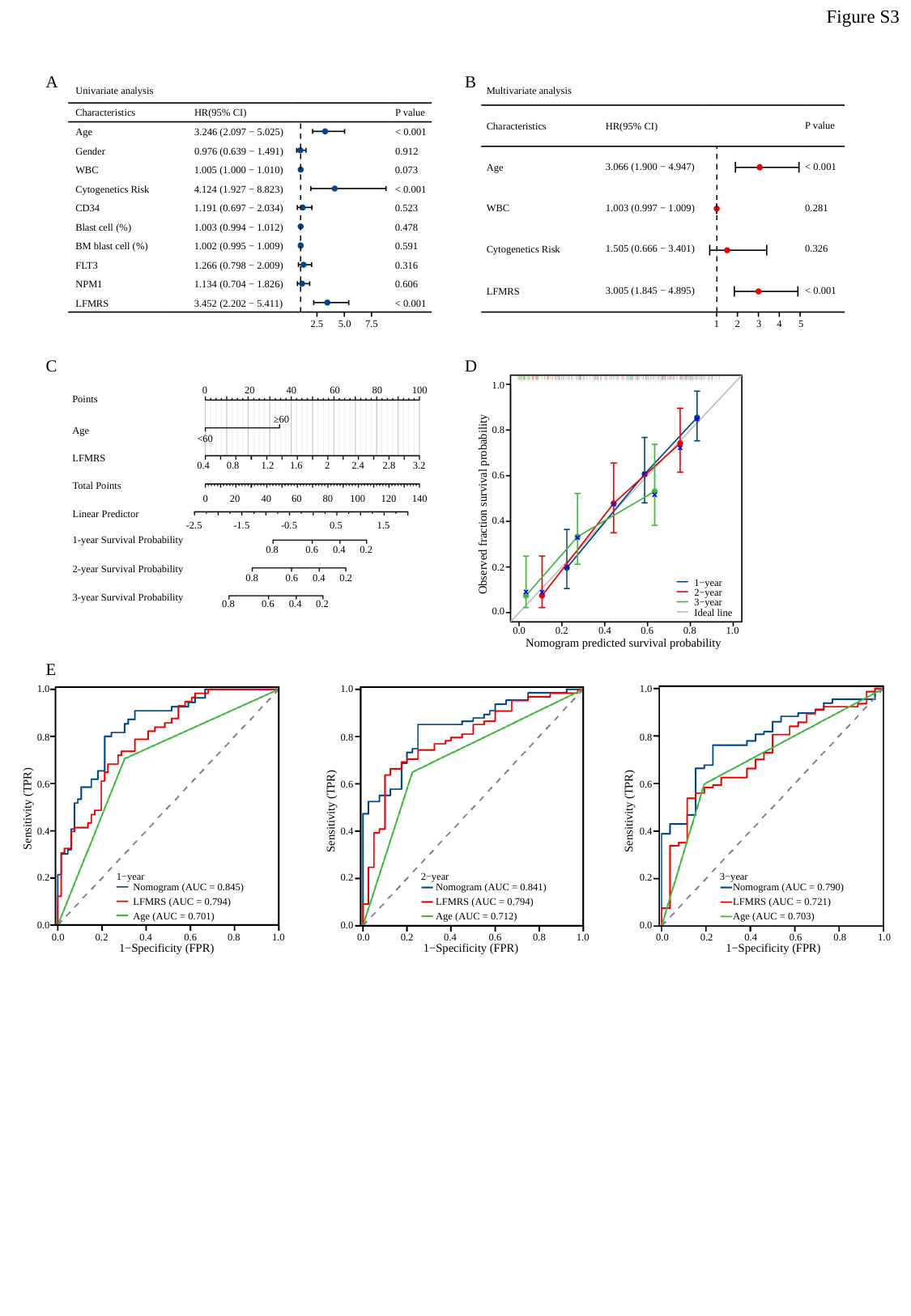

Figure S3
A
B
Univariate analysis
Multivariate analysis
P value
Characteristics
HR(95% CI)
3.066 (1.900 − 4.947)
< 0.001
Age
1.003 (0.997 − 1.009)
0.281
WBC
1.505 (0.666 − 3.401)
0.326
Cytogenetics Risk
3.005 (1.845 − 4.895)
< 0.001
LFMRS
1
2
3
4
5
Characteristics
HR(95% CI)
P value
Age
3.246 (2.097 − 5.025)
< 0.001
Gender
0.976 (0.639 − 1.491)
0.912
WBC
1.005 (1.000 − 1.010)
0.073
Cytogenetics Risk
4.124 (1.927 − 8.823)
< 0.001
CD34
1.191 (0.697 − 2.034)
0.523
Blast cell (%)
1.003 (0.994 − 1.012)
0.478
BM blast cell (%)
1.002 (0.995 − 1.009)
0.591
FLT3
1.266 (0.798 − 2.009)
0.316
NPM1
1.134 (0.704 − 1.826)
0.606
LFMRS
3.452 (2.202 − 5.411)
< 0.001
2.5
5.0
7.5
C
D
1.0
0.8
0.6
Observed fraction survival probability
0.4
0.2
1−year
2−year
3−year
0.0
Ideal line
0.0
0.2
0.4
0.6
0.8
1.0
Nomogram predicted survival probability
0
20
40
60
80
100
Points
≥60
Age
<60
LFMRS
0.4
0.8
1.2
1.6
2
2.4
2.8
3.2
Total Points
0
20
40
60
80
100
120
140
Linear Predictor
-2.5
-1.5
-0.5
0.5
1.5
1-year Survival Probability
0.8
0.6
0.4
0.2
2-year Survival Probability
0.8
0.6
0.4
0.2
3-year Survival Probability
0.8
0.6
0.4
0.2
E
1.0
0.8
0.6
Sensitivity (TPR)
0.4
1−year
0.2
Nomogram (AUC = 0.845)
LFMRS (AUC = 0.794)
Age (AUC = 0.701)
0.0
0.0
0.2
0.4
0.6
0.8
1.0
1−Specificity (FPR)
1.0
0.8
0.6
Sensitivity (TPR)
0.4
2−year
0.2
Nomogram (AUC = 0.841)
LFMRS (AUC = 0.794)
Age (AUC = 0.712)
0.0
0.0
0.2
0.4
0.6
0.8
1.0
1−Specificity (FPR)
1.0
0.8
0.6
Sensitivity (TPR)
0.4
3−year
0.2
Nomogram (AUC = 0.790)
LFMRS (AUC = 0.721)
Age (AUC = 0.703)
0.0
0.0
0.2
0.4
0.6
0.8
1.0
1−Specificity (FPR)

## Slide 4
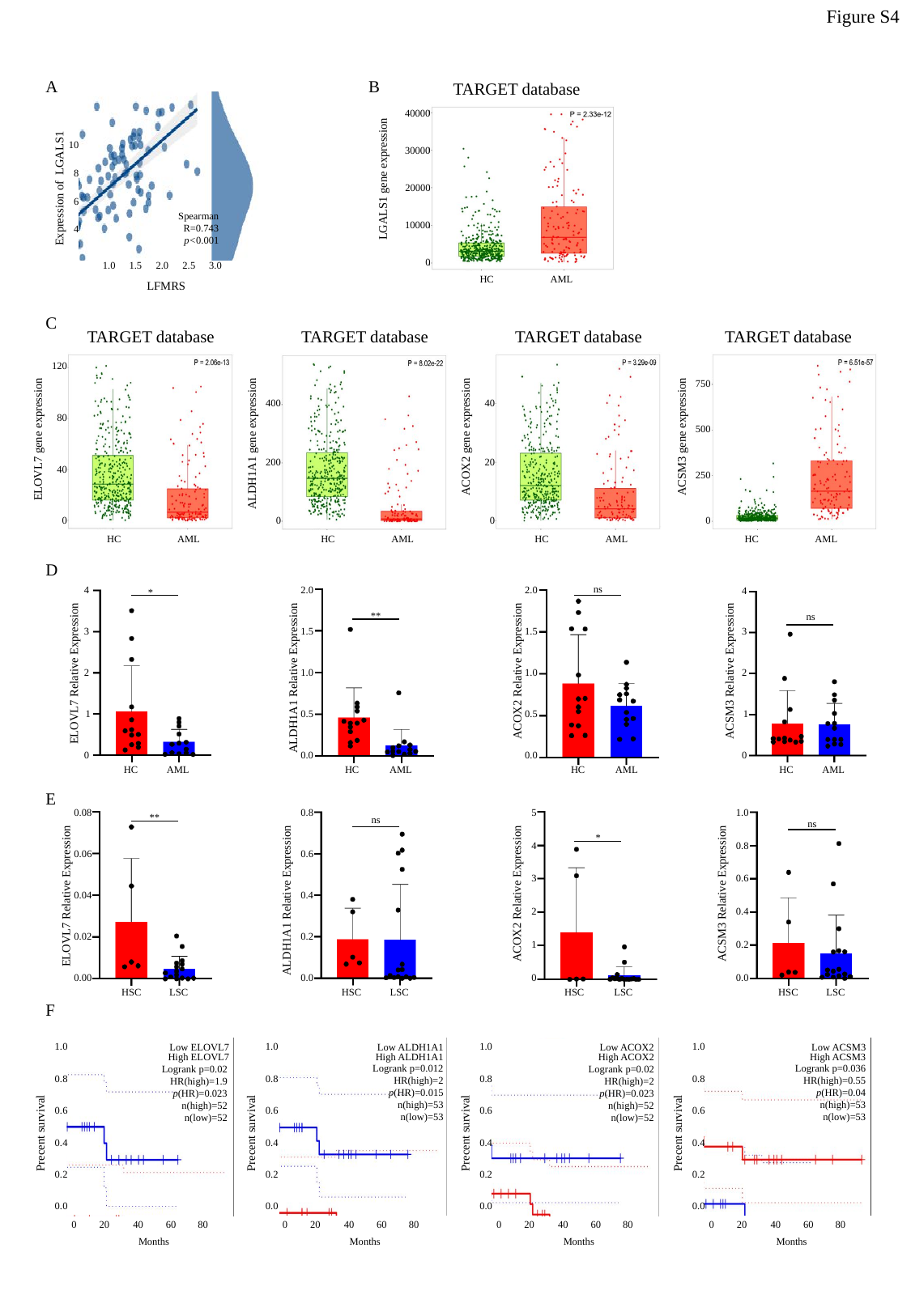

Figure S4
A
B
TARGET database
40000
30000
20000
LGALS1 gene expression
10000
0
10
8
Expression of LGALS1
6
Spearman
R=0.743
p<0.001
4
1.0
1.5
2.0
2.5
3.0
LFMRS
HC
AML
C
TARGET database
120
80
ELOVL7 gene expression
40
0
HC
AML
TARGET database
400
ALDH1A1 gene expression
200
0
HC
AML
TARGET database
40
ACOX2 gene expression
20
0
HC
AML
TARGET database
750
500
ACSM3 gene expression
250
0
HC
AML
D
ns
2.0
1.5
1.0
ACOX2 Relative Expression
0.5
0.0
HC
AML
4
*
3
2
ELOVL7 Relative Expression
1
0
HC
AML
2.0
**
1.5
1.0
ALDH1A1 Relative Expression
0.5
0.0
HC
AML
4
ns
3
2
ACSM3 Relative Expression
1
0
HC
AML
E
0.08
**
0.06
0.04
ELOVL7 Relative Expression
0.02
0.00
HSC
LSC
0.8
ns
0.6
0.4
ALDH1A1 Relative Expression
0.2
0.0
HSC
LSC
5
*
4
3
ACOX2 Relative Expression
2
1
0
HSC
LSC
1.0
ns
0.8
0.6
ACSM3 Relative Expression
0.4
0.2
0.0
HSC
LSC
F
1.0
Low ELOVL7
High ELOVL7
Logrank p=0.02
HR(high)=1.9
p(HR)=0.023
n(high)=52
n(low)=52
0.8
0.6
Precent survival
0.4
0.2
0.0
0
20
40
60
80
Months
1.0
Low ALDH1A1
High ALDH1A1
Logrank p=0.012
HR(high)=2
p(HR)=0.015
n(high)=53
n(low)=53
0.8
0.6
Precent survival
0.4
0.2
0.0
0
20
40
60
80
Months
1.0
Low ACOX2
High ACOX2
Logrank p=0.02
HR(high)=2
p(HR)=0.023
n(high)=52
n(low)=52
0.8
0.6
Precent survival
0.4
0.2
0.0
0
20
40
60
80
Months
1.0
Low ACSM3
High ACSM3
Logrank p=0.036
HR(high)=0.55
p(HR)=0.04
n(high)=53
n(low)=53
0.8
0.6
Precent survival
0.4
0.2
0.0
0
20
40
60
80
Months

## Slide 5
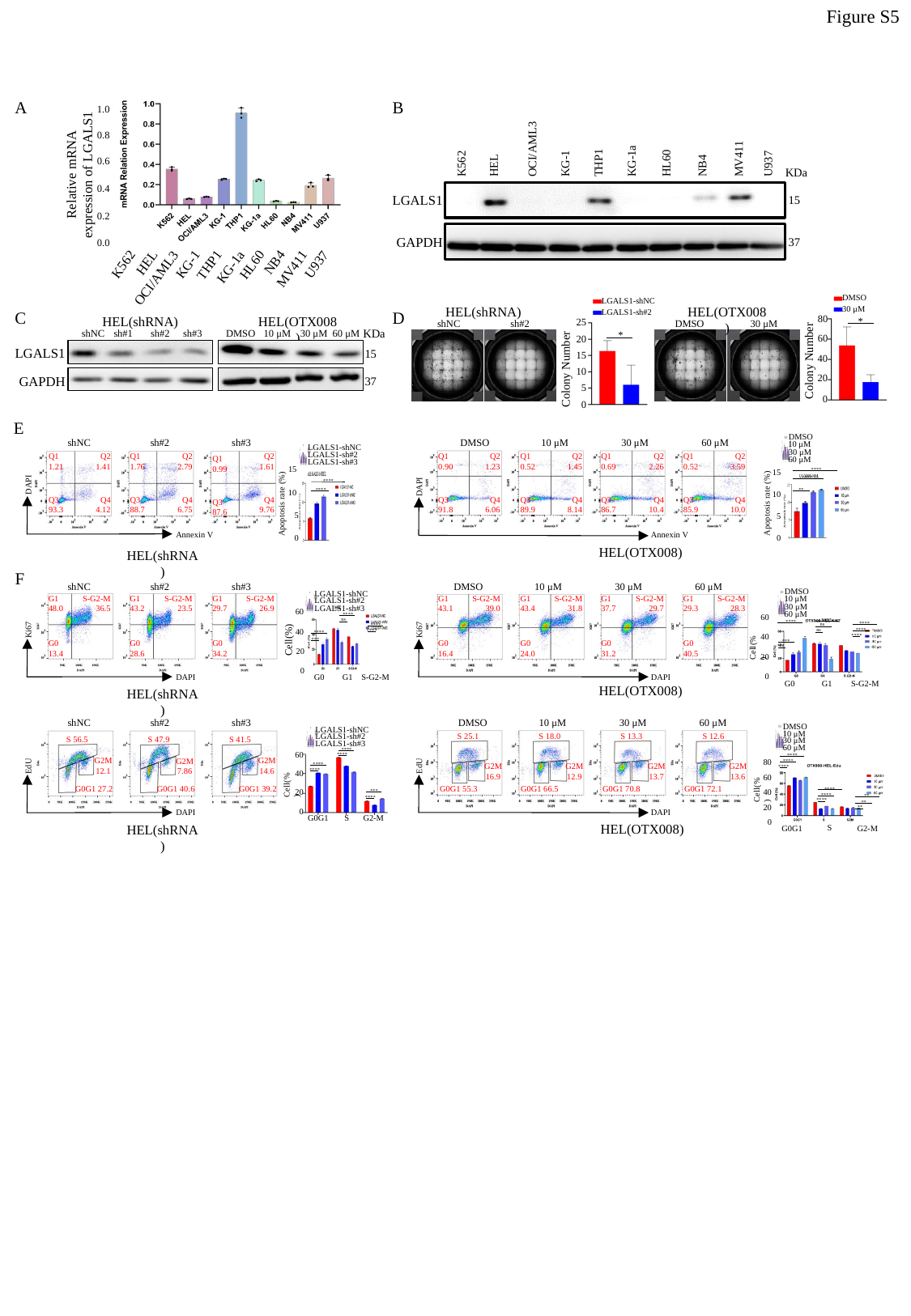

Figure S5
OCI/AML3
KG-1
THP1
KG-1a
HL60
NB4
MV411
U937
K562
HEL
KDa
15
37
1.0
0.8
0.6
Relative mRNA expression of LGALS1
0.4
0.2
0.0
K562
HEL
OCI/AML3
KG-1
THP1
KG-1a
HL60
NB4
MV411
U937
A
B
LGALS1
GAPDH
DMSO
30 μM
80
60
40
Colony Number
20
0
LGALS1-shNC
LGALS1-sh#2
25
*
20
15
Colony Number
10
5
0
HEL(shRNA)
HEL(OTX008)
C
HEL(shRNA)
HEL(OTX008)
KDa
shNC
sh#1
sh#2
sh#3
DMSO
10 μM
30 μM
60 μM
LGALS1
GAPDH
D
*
shNC
sh#2
DMSO
30 μM
15
37
E
DMSO
10 μM
30 μM
60 μM
shNC
sh#2
sh#3
DMSO
10 μM
30 μM
60 μM
LGALS1-shNC
LGALS1-sh#2
LGALS1-sh#3
15
****
****
10
Apoptosis rate (%)
5
0
Q1
1.21
Q2
1.41
Q1
1.76
Q2
2.79
Q2
1.61
Q1
0.90
Q2
1.23
Q1
0.52
Q2
1.45
Q1
0.69
Q2
2.26
Q1
0.52
Q2
3.59
Q1
0.99
DAPI
DAPI
****
15
****
**
10
5
0
Apoptosis rate (%)
Q3
93.3
Q4
4.12
Q3
88.7
Q4
6.75
Q4
9.76
Q3
91.8
Q4
6.06
Q3
89.9
Q4
8.14
Q3
86.7
Q4
10.4
Q3
85.9
Q4
10.0
Q3
87.6
Annexin V
Annexin V
HEL(OTX008)
HEL(shRNA)
F
shNC
sh#2
sh#3
DMSO
10 μM
30 μM
60 μM
DMSO
10 μM
30 μM
60 μM
LGALS1-shNC
LGALS1-sh#2
LGALS1-sh#3
G1
48.0
S-G2-M
36.5
G1
43.2
S-G2-M
23.5
G1
29.7
S-G2-M
26.9
G1
43.1
S-G2-M
39.0
G1
43.4
S-G2-M
31.8
G1
37.7
S-G2-M
29.7
G1
29.3
S-G2-M
28.3
60
****
ns
****
40
****
****
Cell(%)
***
20
0
G0
G1
S-G2-M
Ki67
Ki67
60
****
****
****
ns
****
ns
40
****
***
Cell(%)
**
20
0
G0
G1
S-G2-M
G0
13.4
G0
28.6
G0
34.2
G0
16.4
G0
24.0
G0
31.2
G0
40.5
DAPI
DAPI
HEL(OTX008)
HEL(shRNA)
shNC
sh#2
sh#3
DMSO
10 μM
30 μM
60 μM
DMSO
10 μM
30 μM
60 μM
LGALS1-shNC
LGALS1-sh#2
LGALS1-sh#3
S 25.1
S 18.0
S 13.3
S 12.6
S 56.5
S 47.9
S 41.5
EdU
EdU
****
60
****
****
G2M
12.1
G2M
14.6
G2M
7.86
80
****
****
G2M
16.9
G2M
12.9
G2M
13.7
G2M
13.6
****
****
40
60
Cell(%)
G0G1 27.2
G0G1 40.6
G0G1 39.2
Cell(%)
G0G1 55.3
G0G1 66.5
G0G1 70.8
G0G1 72.1
****
40
20
***
****
**
****
****
**
20
**
0
DAPI
DAPI
G0G1
S
G2-M
0
HEL(OTX008)
HEL(shRNA)
S
G0G1
G2-M

## Slide 6
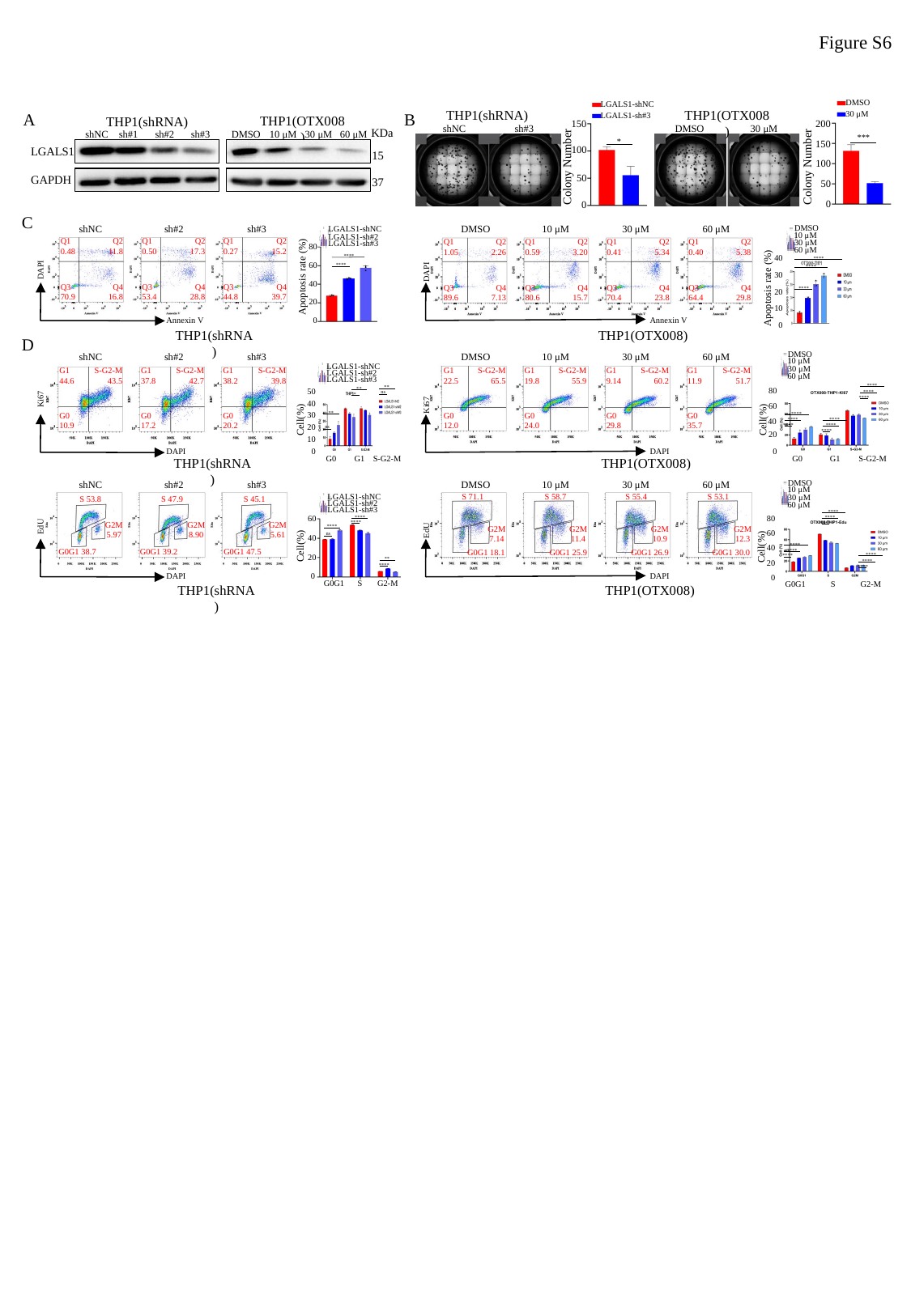

Figure S6
DMSO
30 μM
200
150
100
Colony Number
50
0
LGALS1-shNC
LGALS1-sh#3
150
100
50
0
Colony Number
THP1(shRNA)
THP1(OTX008)
A
THP1(OTX008)
THP1(shRNA)
DMSO
10 μM
30 μM
60 μM
shNC
sh#1
sh#2
sh#3
LGALS1
GAPDH
B
shNC
sh#3
DMSO
30 μM
KDa
***
*
15
37
C
shNC
sh#2
sh#3
DMSO
10 μM
30 μM
60 μM
DMSO
10 μM
30 μM
60 μM
LGALS1-shNC
LGALS1-sh#2
LGALS1-sh#3
80
****
60
****
40
20
0
Apoptosis rate (%)
Q1
0.48
Q2
11.8
Q1
0.50
Q2
17.3
Q1
0.27
Q2
15.2
Q1
1.05
Q2
2.26
Q1
0.59
Q2
3.20
Q1
0.41
Q2
5.34
Q1
0.40
Q2
5.38
40
****
****
30
Apoptosis rate (%)
****
20
10
0
DAPI
DAPI
Q3
70.9
Q4
16.8
Q3
53.4
Q4
28.8
Q3
44.8
Q4
39.7
Q3
89.6
Q4
7.13
Q3
80.6
Q4
15.7
Q3
70.4
Q4
23.8
Q3
64.4
Q4
29.8
Annexin V
Annexin V
THP1(shRNA)
THP1(OTX008)
D
DMSO
10 μM
30 μM
60 μM
shNC
sh#2
sh#3
DMSO
10 μM
30 μM
60 μM
LGALS1-shNC
LGALS1-sh#2
LGALS1-sh#3
G1
44.6
S-G2-M
43.5
G1
37.8
S-G2-M
42.7
G1
38.2
S-G2-M
39.8
G1
22.5
S-G2-M
65.5
G1
19.8
S-G2-M
55.9
G1
9.14
S-G2-M
60.2
G1
11.9
S-G2-M
51.7
****
Ki67
**
80
**
50
****
ns
*
Ki67
****
40
60
**
Cell(%)
30
G0
10.9
G0
17.2
G0
20.2
G0
12.0
G0
24.0
G0
29.8
G0
35.7
Cell(%)
****
40
****
****
****
20
****
ns
****
20
10
DAPI
DAPI
0
0
G0
G1
S-G2-M
G0
G1
S-G2-M
THP1(shRNA)
THP1(OTX008)
DMSO
10 μM
30 μM
60 μM
shNC
sh#2
sh#3
DMSO
10 μM
30 μM
60 μM
LGALS1-shNC
LGALS1-sh#2
LGALS1-sh#3
S 71.1
S 58.7
S 55.4
S 53.1
S 53.8
S 47.9
S 45.1
EdU
****
80
****
****
60
Cell(%)
****
40
****
****
****
****
20
****
0
G0G1
S
G2-M
60
****
****
G2M
5.97
G2M
8.90
G2M
5.61
EdU
****
G2M
7.14
G2M
11.4
G2M
10.9
G2M
12.3
ns
40
Cell(%)
G0G1 38.7
G0G1 39.2
G0G1 47.5
G0G1 18.1
G0G1 25.9
G0G1 26.9
G0G1 30.0
20
**
****
DAPI
DAPI
0
G0G1
S
G2-M
THP1(shRNA)
THP1(OTX008)

## Slide 7
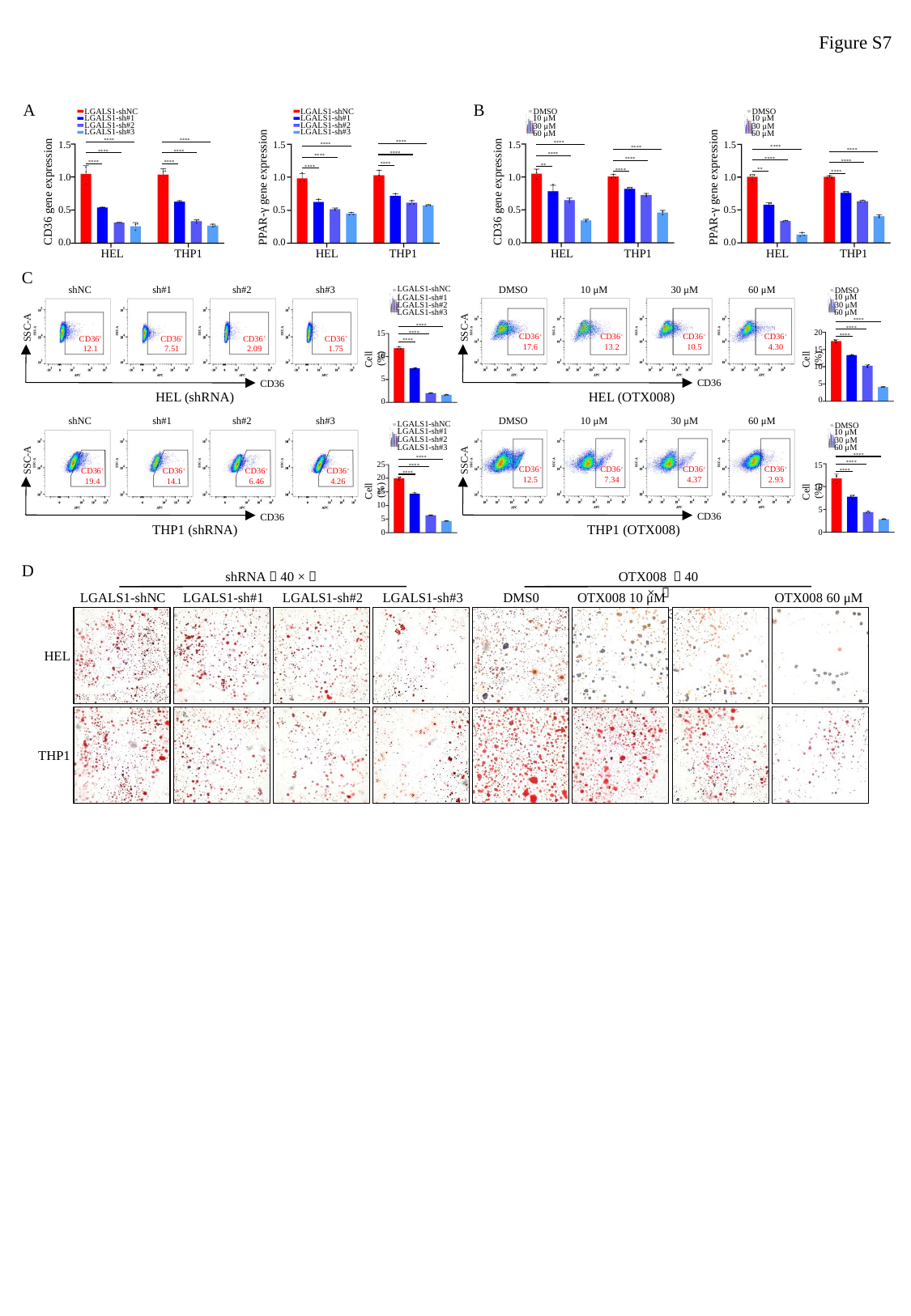

Figure S7
A
B
LGALS1-shNC
LGALS1-sh#1
LGALS1-sh#2
LGALS1-sh#3
LGALS1-shNC
LGALS1-sh#1
LGALS1-sh#2
LGALS1-sh#3
DMSO
10 μM
30 μM
60 μM
DMSO
10 μM
30 μM
60 μM
****
****
****
****
****
****
1.5
1.0
CD36 gene expression
0.5
0.0
HEL
THP1
1.5
****
****
****
****
****
****
1.0
PPAR-γ gene expression
0.5
0.0
HEL
THP1
1.5
****
****
**
****
****
****
1.0
CD36 gene expression
0.5
0.0
HEL
THP1
1.5
****
****
**
****
****
****
1.0
PPAR-γ gene expression
0.5
0.0
HEL
THP1
C
LGALS1-shNC
LGALS1-sh#1
LGALS1-sh#2
LGALS1-sh#3
shNC
sh#1
sh#2
sh#3
DMSO
10 μM
30 μM
60 μM
DMSO
10 μM
30 μM
60 μM
SSC-A
CD36
HEL (shRNA)
SSC-A
CD36
HEL (OTX008)
****
****
****
20
15
****
CD36+
17.6
CD36+
13.2
CD36+
10.5
CD36+
4.30
****
CD36+
12.1
CD36+
7.51
CD36+
2.09
CD36+
1.75
****
15
10
Cell (%)
Cell (%)
10
5
5
0
0
shNC
sh#1
sh#2
sh#3
DMSO
10 μM
30 μM
60 μM
LGALS1-shNC
LGALS1-sh#1
LGALS1-sh#2
LGALS1-sh#3
DMSO
10 μM
30 μM
60 μM
SSC-A
CD36
THP1 (shRNA)
SSC-A
CD36
THP1 (OTX008)
****
****
25
****
15
****
CD36+
12.5
CD36+
7.34
CD36+
4.37
CD36+
2.93
CD36+
19.4
CD36+
14.1
CD36+
6.46
CD36+
4.26
****
****
20
10
15
Cell (%)
Cell (%)
10
5
5
0
0
D
shRNA（40 ×）
OTX008 （40 × ）
LGALS1-shNC
LGALS1-sh#1
LGALS1-sh#2
LGALS1-sh#3
DMS0
OTX008 10 μM
	OTX008 30 μM
OTX008 60 μM
HEL
THP1

## Slide 8
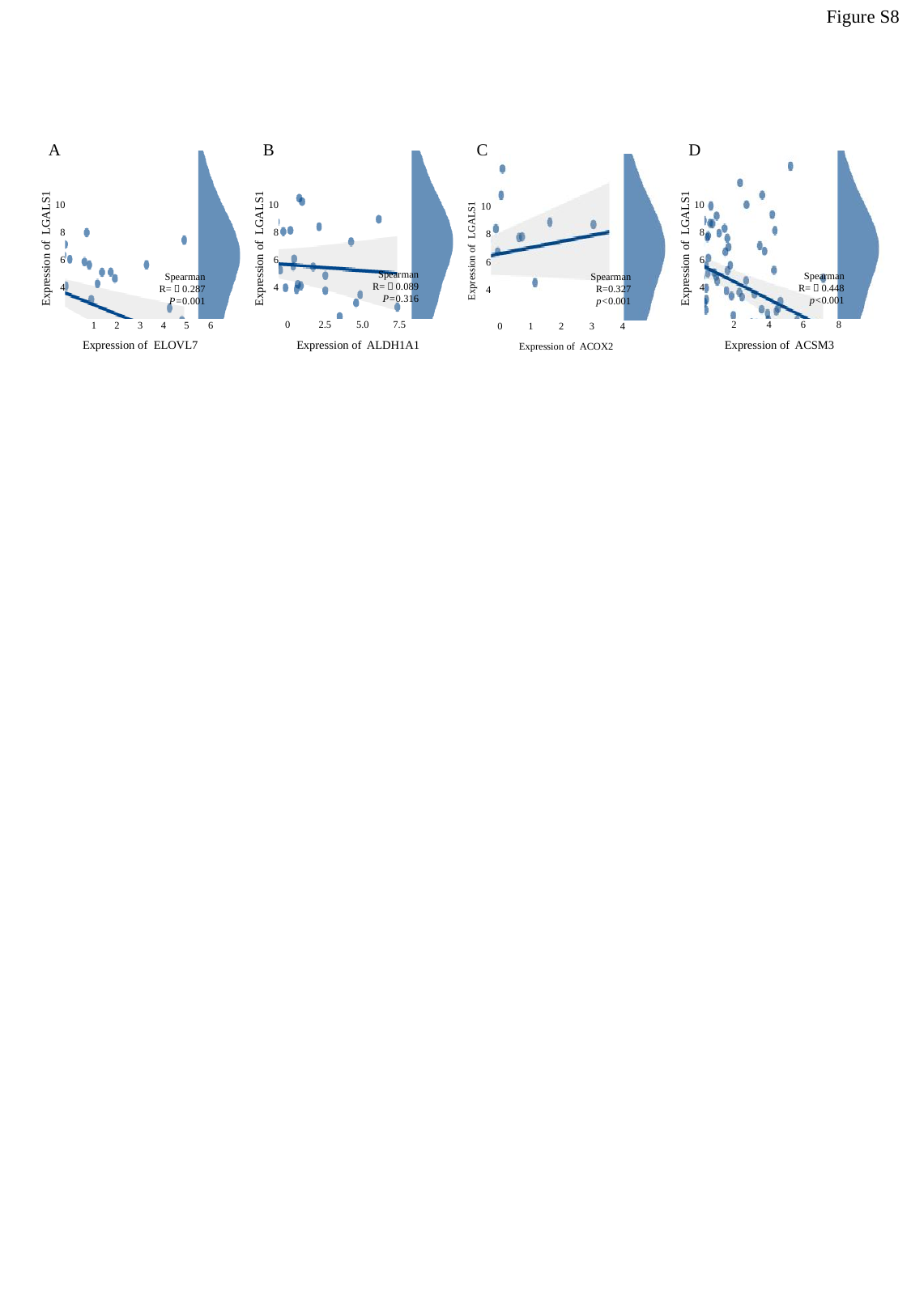

Figure S8
A
B
C
D
10
8
Expression of LGALS1
6
Spearman
R=－0.287
P=0.001
4
1
2
3
4
5
6
Expression of ELOVL7
10
8
Expression of LGALS1
6
Spearman
R=－0.089
P=0.316
4
0
2.5
5.0
7.5
Expression of ALDH1A1
10
8
Expression of LGALS1
6
Spearman
R=－0.448
p<0.001
4
2
4
6
8
Expression of ACSM3
10
8
Expression of LGALS1
6
Spearman
R=0.327
p<0.001
4
0
1
2
3
4
Expression of ACOX2
