# Supplementary material for: A novel LGALS1-depended and immune-associated fatty acid metabolism risk model in acute myeloid leukemia stem cells
Source: Cell Death Dis. 2024 Jul 5;15(7):482. doi: 10.1038/s41419-024-06865-6 (PMC11224233; doi:10.1038/s41419-024-06865-6)
Supplement: Supplementary file 2 — sup figure legends [file 41419_2024_6865_MOESM2_ESM.docx]

**Supplementary Figure Legend**

**Figure S1. Related to Figure 1.**

(A) The fatty acid metabolism-related genes were screened out from Hallmark, KEGG, Reactom, and WP databases. (B) The three datasets (GSE68172, GSE17054, GSE24395) were selected to screen the differential expression genes between HSCs and LSCs. (C-F) Consensus CDF (C), Delta area (D), tracking plot (E), PCA plot (F) for validation of the clustering results. (G-J) The proportion of overall survival (G), FAB subtypes (H), WBC counts (I), cytogenetics risks (J) among different clusters. (K) Partial likelihood deviation for different number of variables. **p* < 0.05; ***p* < 0.01; ****p* < 0.001; *****p* < 0.0001; ns, not significant.

**Figure S2. Related to Figure 2.**

(A) Ranked dot and scatter plots of the model gene expressions in the TCGA cohort. (B-E) The 1-, 2- and 3-year ROC curves of the LFMRS in the BeatAML (B), GSE71014 (C), GSE12417 (D), and GSE37642 (E) cohorts. (F) Volcano plots showed the correlation of LFMRS with IC50 values of drugs. (G) Correlation between LFMRS and ATRA. *p < 0.05; **p < 0.01; ***p < 0.001; ****p < 0.0001; ns, not significant.

**Figure S3. Key factors of LFMRS and age affecting the prognosis of patients with AML.**

(A) Univariate COX regression analysis of clinical characteristics and LFMRS. (B) Multivariate COX regression analysis of clinical characteristics and LFMRS. (C) Nomogram combining LFMRS with age. (D) Calibration plots for predicting 1 -, 2 -, 3-year OS of AML patients. (E) The 1-, 2- and 3-year ROC curves for prediction of survival by the nomogram, LFMRS and age. *p < 0.05; **p < 0.01; ***p < 0.001; ****p < 0.0001; ns, not significant.

**Figure S4. Related to Figure 3.**

(A) The correlation of LGALS1 expression and LFMRS in TCGA database. LFMRS = LGALS1 * 0.260 + ELOVL7 * 0.215 + ALDH1A1 * 0.045 + ACOX2 * 0.013 - ACSM3 * 0.138. (B) The transcript levels of LGALS1 in AML samples compared with that in healthy individuals were identified from TARGET database. (C) The transcript levels of ELOVL7, ALDH1A1, ACOX2, and ACSM3 in AML samples compared with that in healthy individuals were identified from TARGET database. (D) The mRNA levels of ELOVL7, ALDH1A1, ACOX2, and ACSM3 in primary AML cases (n=13, AML#1-AML#13) and healthy control cases (n=13). (E) The mRNA levels of ELOVL7, ALDH1A1, ACOX2, and ACSM3 in HSCs and LSCs from healthy control cases and AML cases, respectively (LSCs from AML#1-AML#16). (F) Kaplan-Meier plots of overall survival in TCGA cohorts for AML patients, stratified on the basis of ELOVL7, ALDH1A1, ACOX2, and ACSM3 expression above or below the median, respectively. **p* < 0.05; ***p* < 0.01; ****p* < 0.001; *****p* < 0.0001; ns, not significant.

**Figure S5. LGALS1 promotes cell proliferation and inhibits cell apoptosis of leukemia cells.**

(A-B) Endogenous LGALS1 expression was determined in various AML cells in qRT-PCR (A) and western blotting (B). (C) Efficiencies of LGALS1 silence in HEL cells were determined by western blotting. (D) Cell growth was determined by colony formation assay under a light microscope, and the percentage of colony formation units were shown. (E) Cell apoptosis was determined by flow cytometric analysis. (F) Cell cycle distribution was detected by flow cytometric analysis *via* Ki67 staining (upper) and EdU staining (lower), respectively, and the bar graph showed the percentage of G0/G1, S, and G2/M phase cells.

**Figure S6. Continue to Figure S5.**

(A) Efficiencies of LGALS1 silence in THP1 cells were determined by western blotting. (B) Cell growth was determined by colony formation assay under a light microscope, and the percentage of colony formation units were shown. (C) Cell apoptosis was determined by flow cytometric analysis. (D) Cell cycle distribution was detected by flow cytometric analysis via Ki67 staining (upper) and EdU staining (lower), respectively, and the bar graph showed the percentage of G0/G1, S, and G2/M phase cells. *p < 0.05; **p < 0.01; ***p < 0.001; ****p < 0.0001; ns, not significant.

**Figure S7. LGALS1 plays a key role in lipid metabolism reprogramming of leukemia cells.**

(A-D) HEL and THP1 transfected with shRNA against GALS1, or treated with DMSO or OTX008 were cultured. (A-B) The mRNA levels of CD36 and PPAR-γ were detected by qRT-PCR. (C) The protein levels of CD36 were determined by FCM using anti-CD36-APC (1:100, BioLegend, America). (D) Representative images of Oil Red O staining. **p* < 0.05; ***p* < 0.01; ****p* < 0.001; *****p* < 0.0001; ns, not significant.

**Figure S8. The correlation analysis of LGALS1 and others members of LFMRS**

(A-D) The correlation of LGALS1 expression and ELOVL7 (A), ALDH1A1 (B), ACOX2 (C), and ACSM3 (D) in TCGA database. **p* < 0.05; ***p* < 0.01; ****p* < 0.001; *****p* < 0.0001; ns, not significant.
