# Supplementary material for: A novel LGALS1-depended and immune-associated fatty acid metabolism risk model in acute myeloid leukemia stem cells
Source: Cell Death Dis. 2024 Jul 5;15(7):482. doi: 10.1038/s41419-024-06865-6 (PMC11224233; doi:10.1038/s41419-024-06865-6)
Supplement: Supplementary file 3 — CDDIS-24-0762RR WB daw data [file 41419_2024_6865_MOESM3_ESM.pptx]

## Slide 1
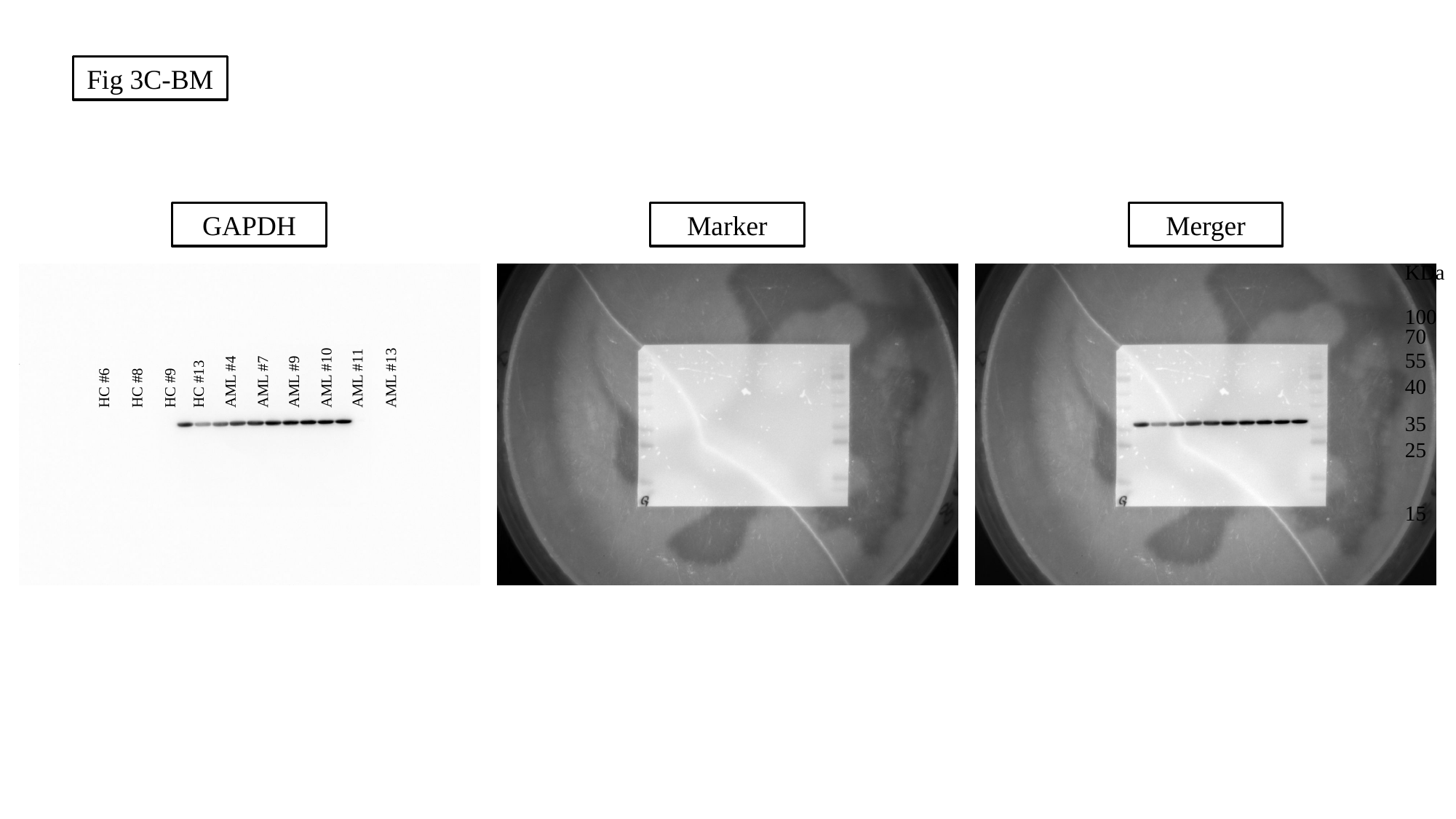

Fig 3C-BM
GAPDH
Marker
Merger
KDa
100
70
55
HC #8
AML #4
AML #7
AML #9
AML #10
AML #11
AML #13
HC #9
HC #13
HC #6
40
35
25
15

## Slide 2
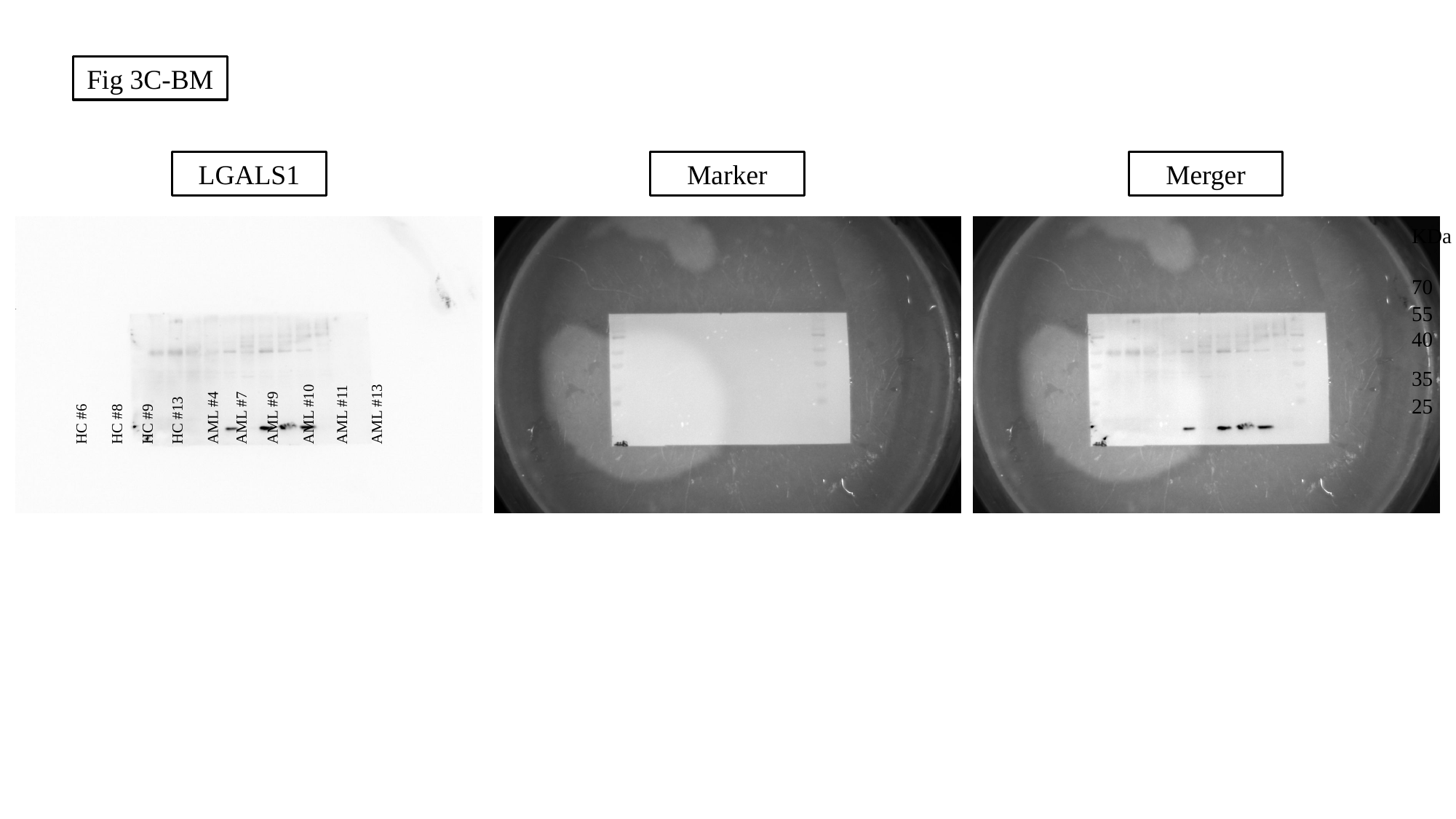

Fig 3C-BM
LGALS1
Marker
Merger
KDa
70
55
40
35
25
HC #8
AML #4
AML #7
AML #9
AML #10
AML #11
AML #13
HC #9
HC #13
HC #6

## Slide 3
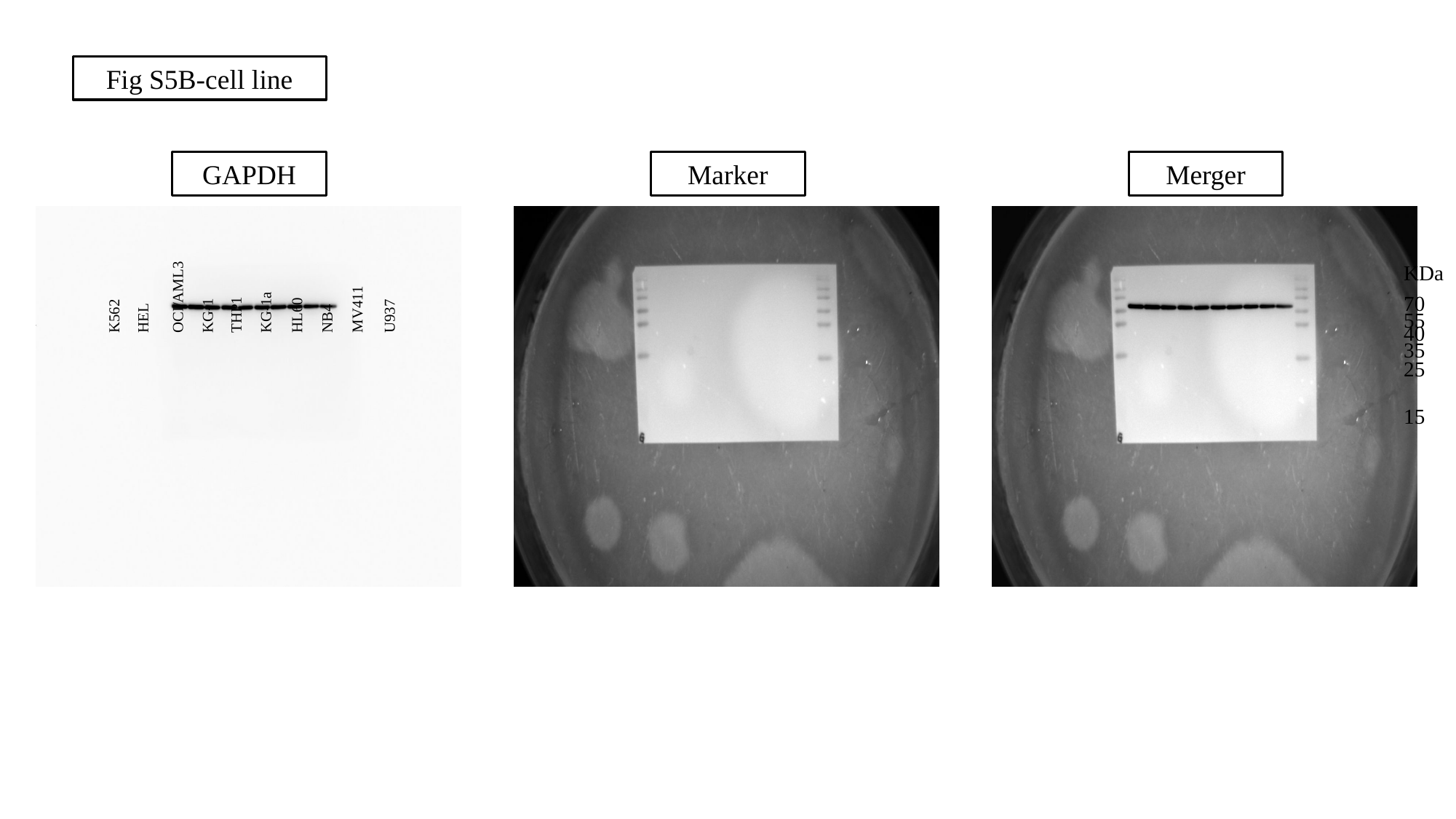

Fig S5B-cell line
GAPDH
Marker
Merger
KDa
OCI/AML3
KG-1
THP1
KG-1a
HL60
NB4
MV411
U937
70
K562
HEL
55
40
35
25
15

## Slide 4
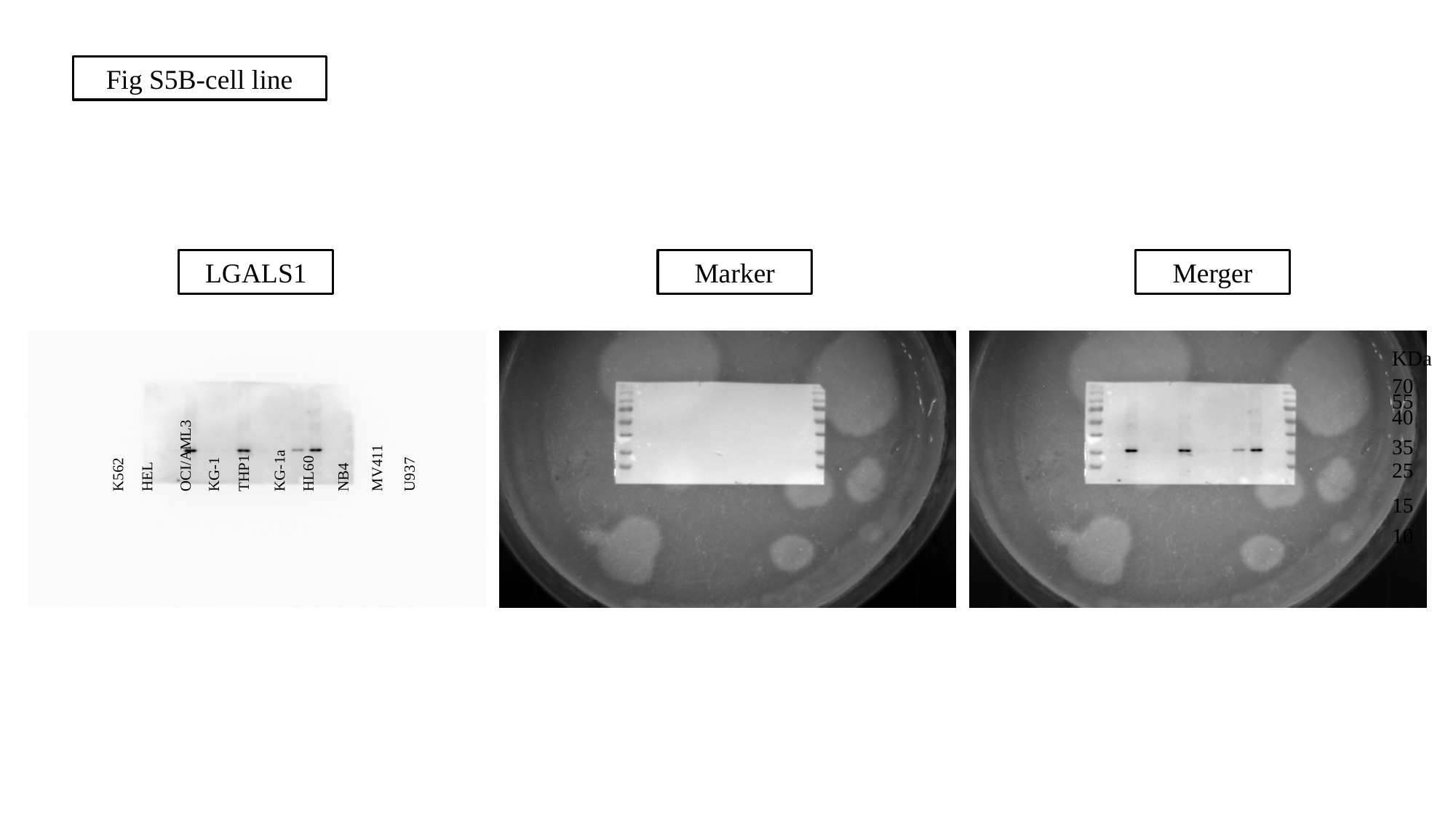

Fig S5B-cell line
LGALS1
Marker
Merger
KDa
70
55
40
35
OCI/AML3
KG-1
THP1
KG-1a
HL60
NB4
MV411
U937
K562
HEL
25
15
10

## Slide 5
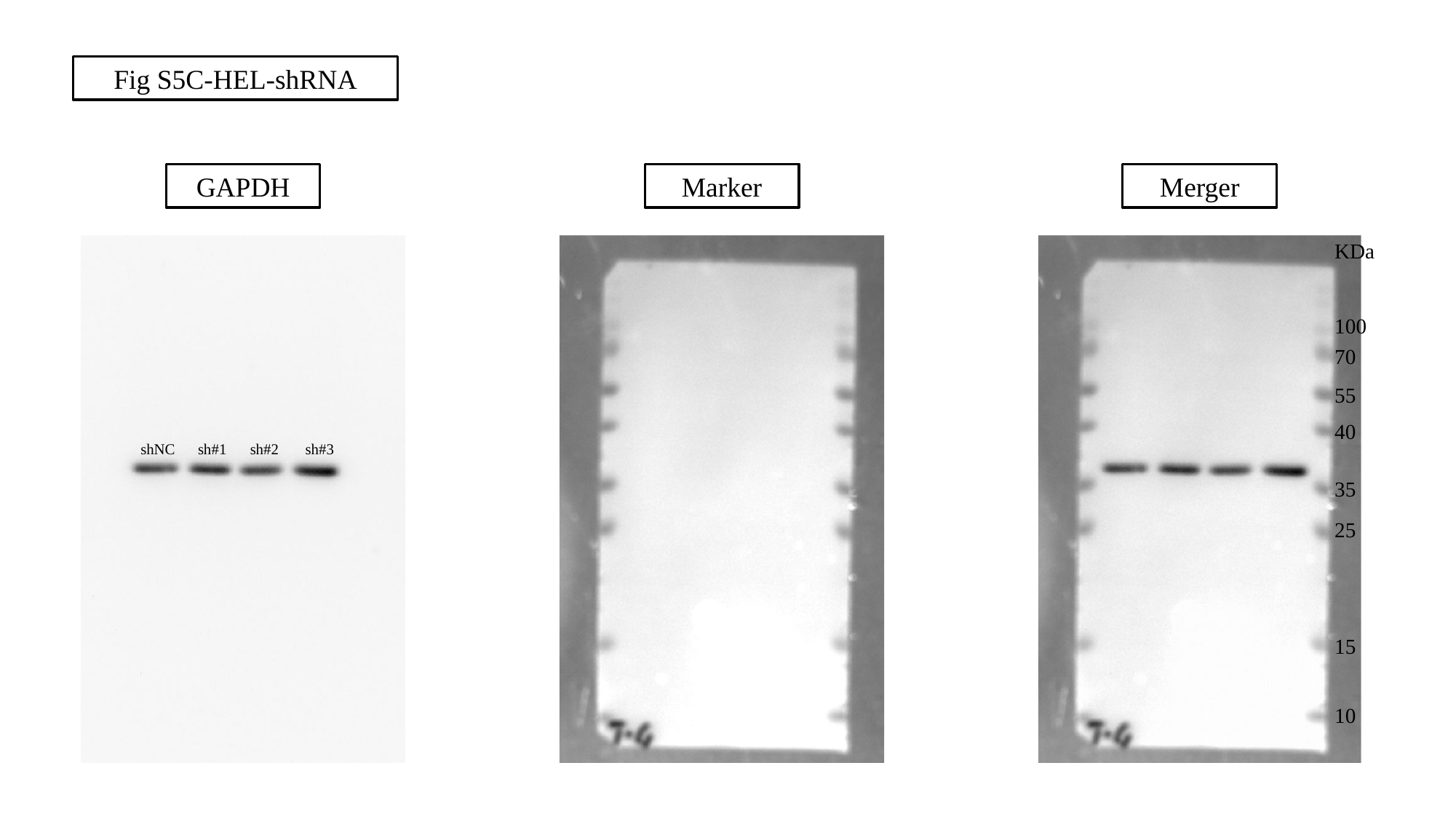

Fig S5C-HEL-shRNA
GAPDH
Marker
Merger
KDa
100
70
55
40
shNC
sh#1
sh#2
sh#3
shNC
sh#1
sh#2
sh#3
35
25
15
10

## Slide 6
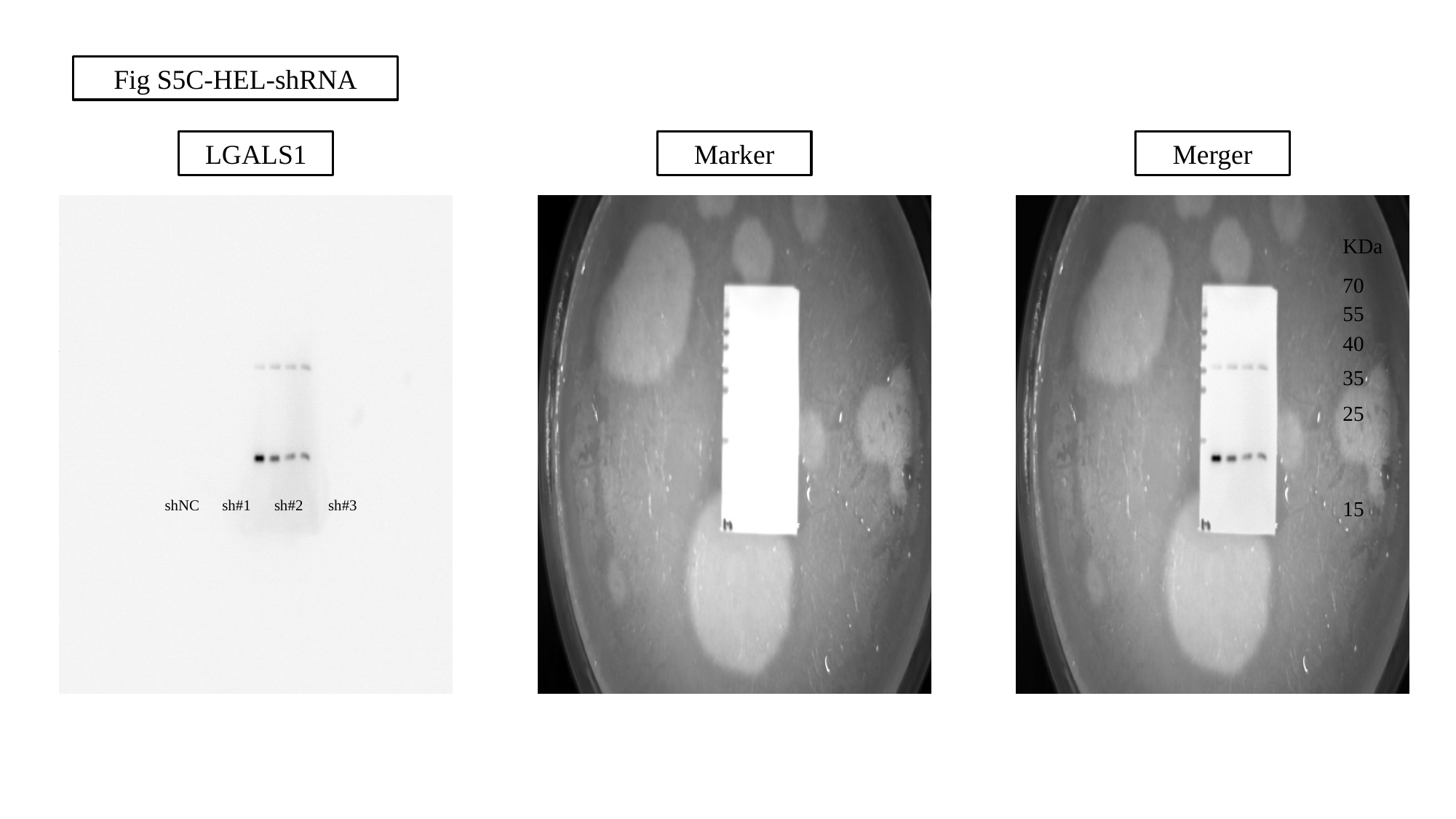

Fig S5C-HEL-shRNA
LGALS1
Marker
Merger
KDa
70
55
40
35
25
shNC
sh#1
sh#2
sh#3
15

## Slide 7
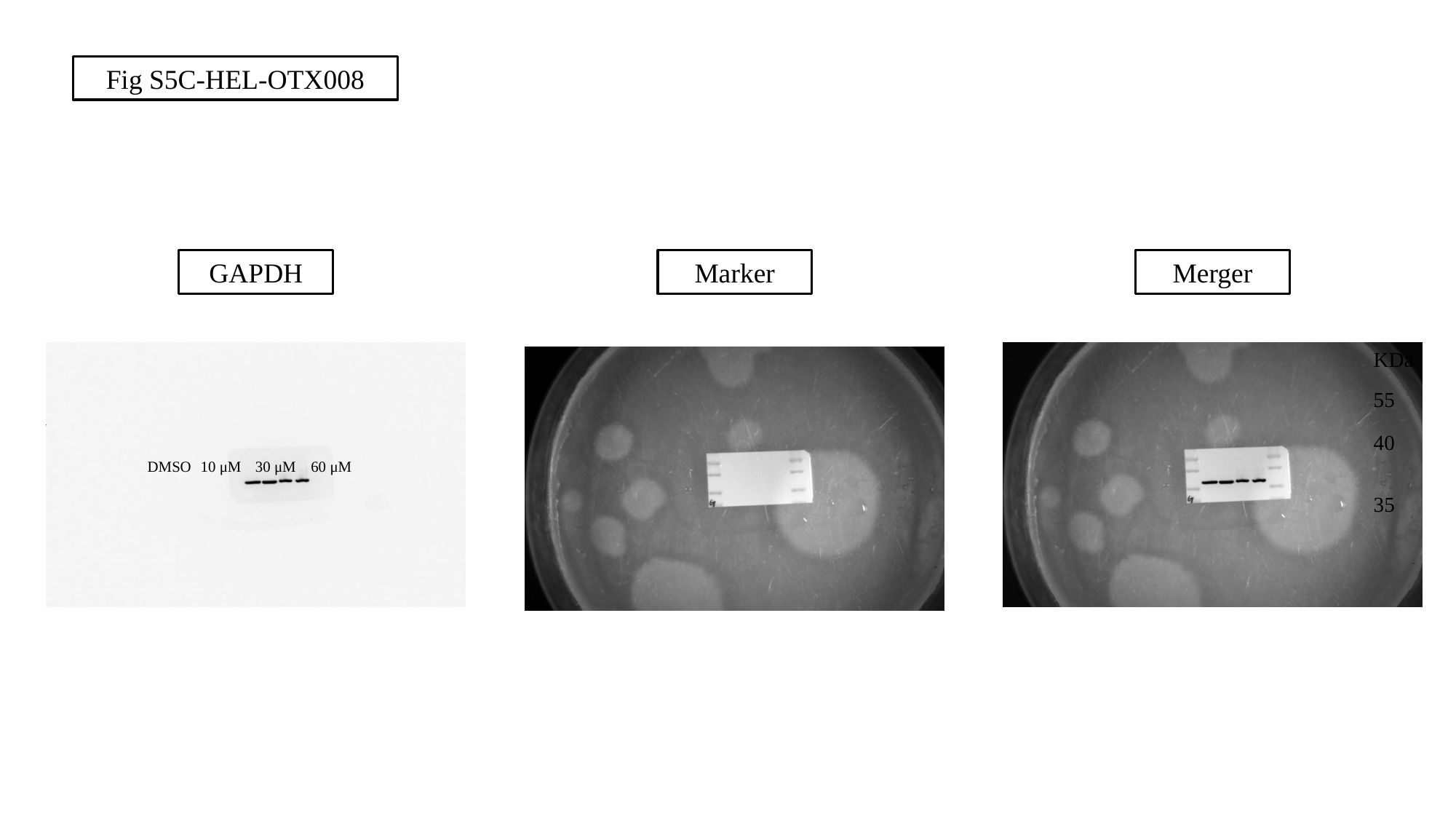

Fig S5C-HEL-OTX008
GAPDH
Marker
Merger
KDa
55
40
DMSO
10 μM
30 μM
60 μM
35

## Slide 8
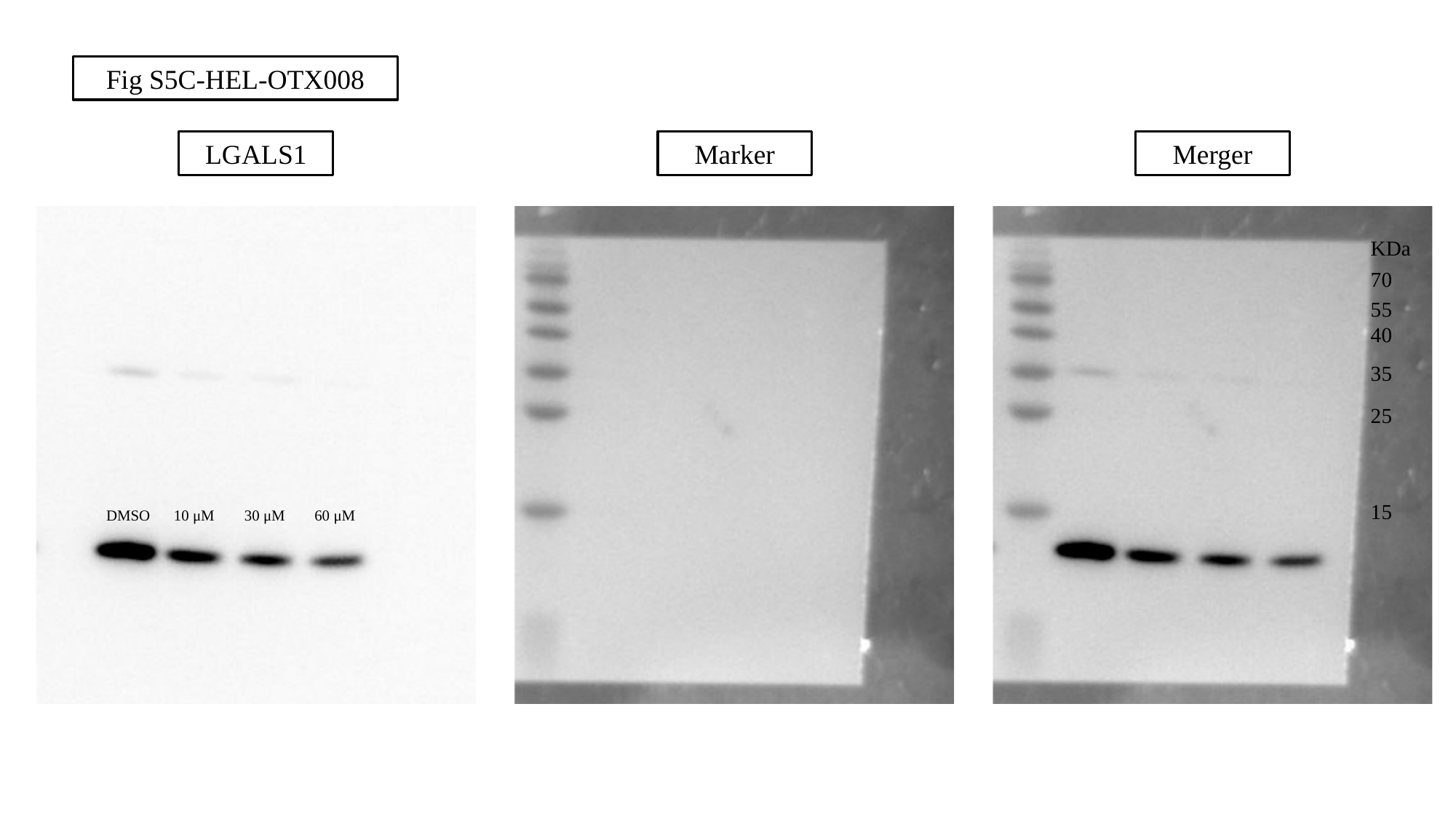

Fig S5C-HEL-OTX008
LGALS1
Marker
Merger
KDa
70
55
40
35
25
15
DMSO
10 μM
30 μM
60 μM

## Slide 9
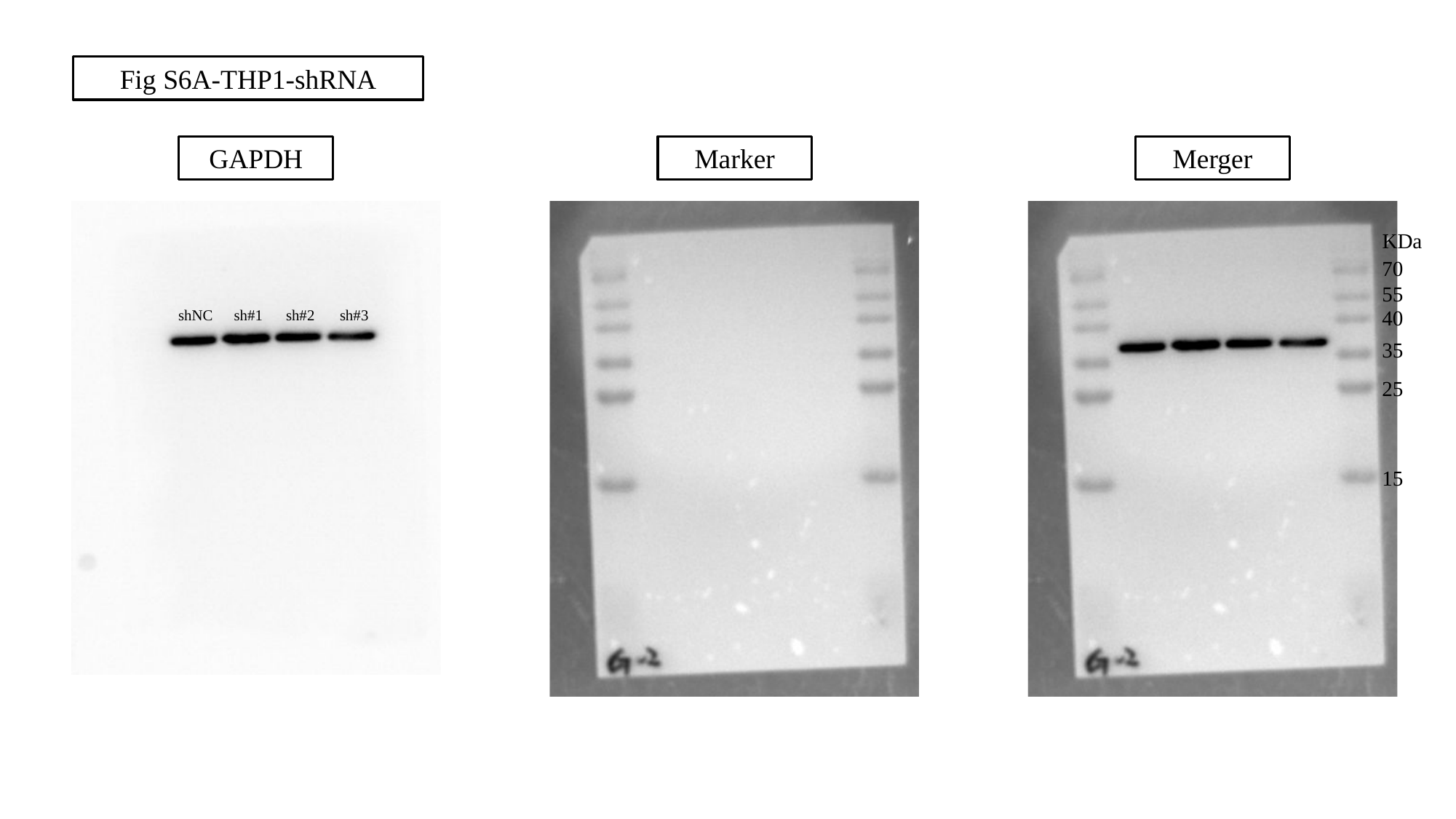

Fig S6A-THP1-shRNA
GAPDH
Marker
Merger
KDa
70
55
40
shNC
sh#1
sh#2
sh#3
35
25
15

## Slide 10
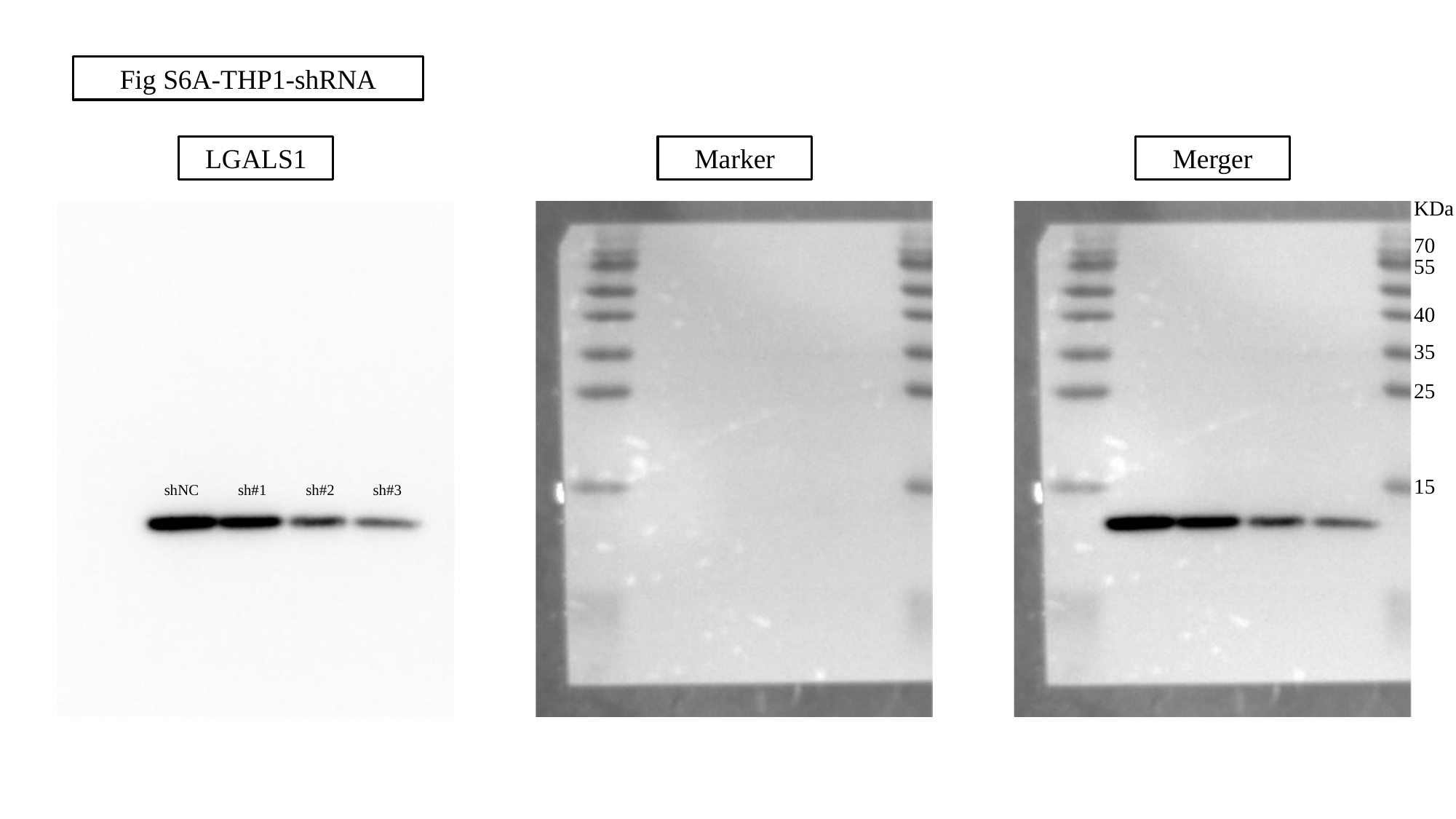

Fig S6A-THP1-shRNA
LGALS1
Marker
Merger
KDa
70
55
40
35
25
15
shNC
sh#1
sh#2
sh#3

## Slide 11
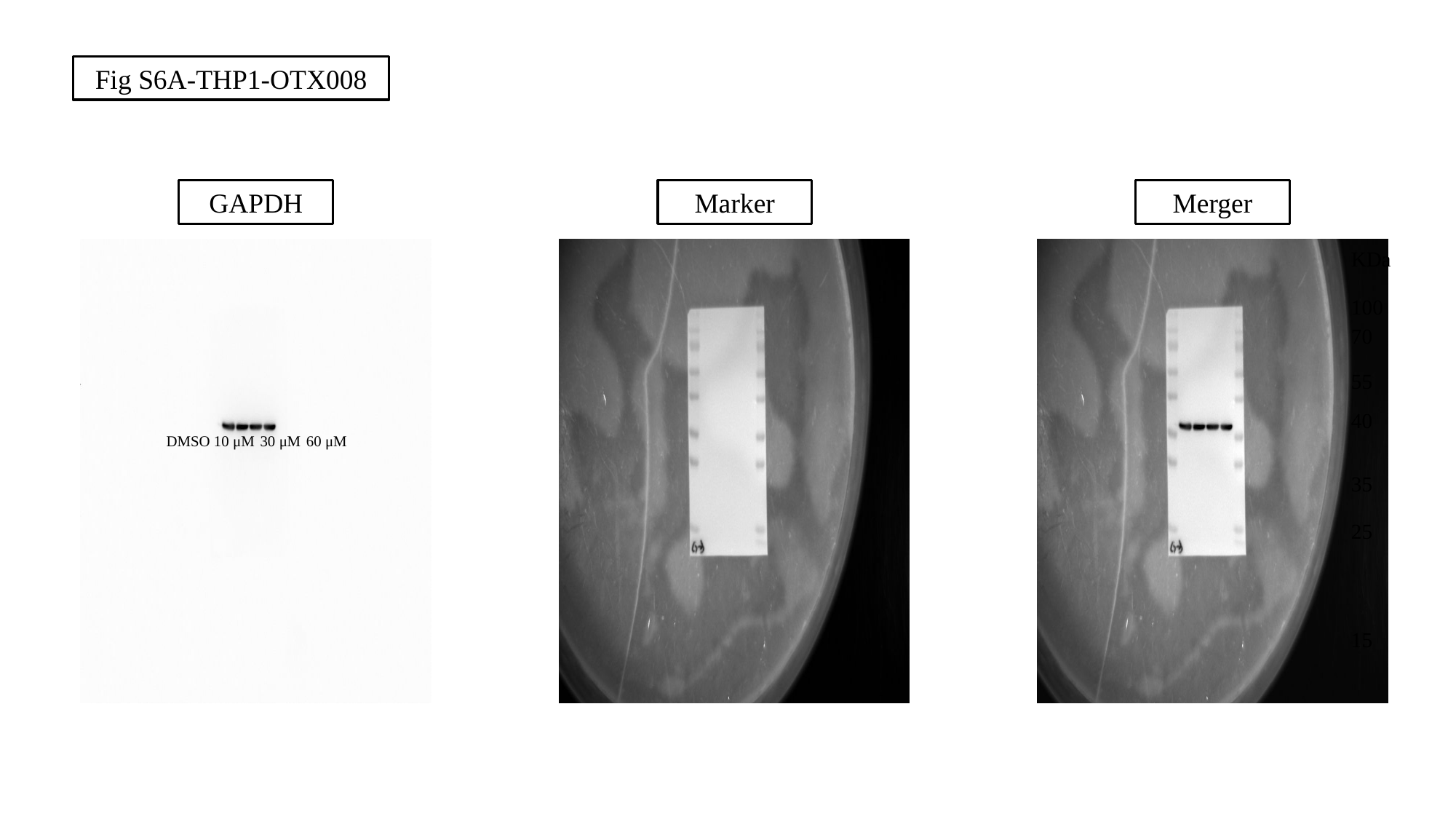

Fig S6A-THP1-OTX008
GAPDH
Marker
Merger
KDa
100
70
55
40
DMSO
10 μM
30 μM
60 μM
35
25
15

## Slide 12
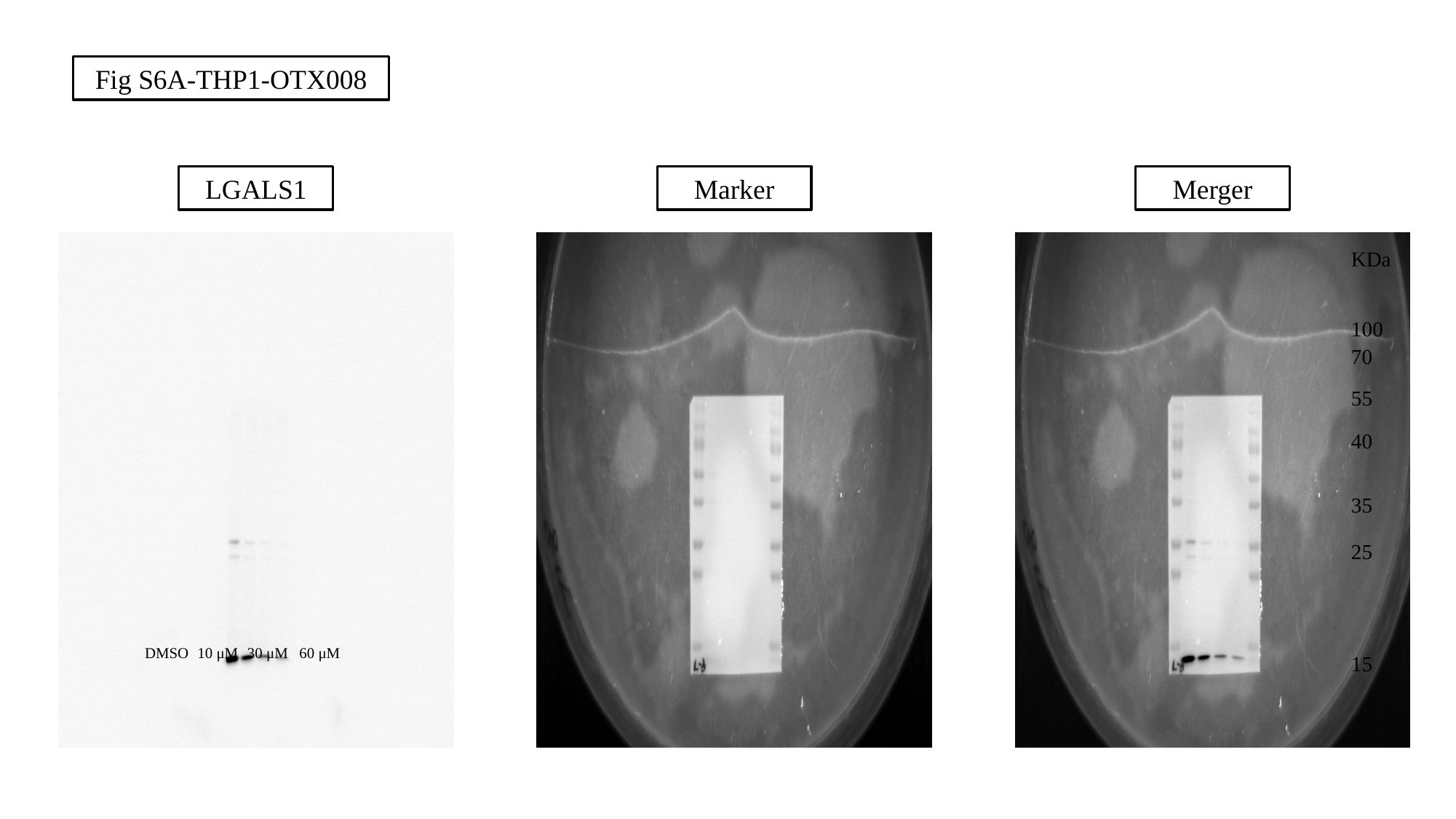

Fig S6A-THP1-OTX008
LGALS1
Marker
Merger
KDa
100
70
55
40
35
25
DMSO
10 μM
30 μM
60 μM
15
